# Engineering of tissue in microphysiological systems demonstrated by modelling skeletal muscle

**DOI:** 10.1093/rb/rbaf059

**Published:** 2025-06-16

**Authors:** Yuan Gao, Zilin Zhang, Yu Yao, Jing Zhang, Xiaoran Li, Keyu Yang, Nuo Si, Zaozao Chen, Zhongze Gu, Ningbei Yin

**Affiliations:** Plastic Surgery Hospital, Chinese Academy of Medical Sciences and Peking Union Medical College, Beijing 100043, China; State Key Laboratory of Bioelectronics, School of Biological Science and Medical Engineering, Southeast University, Nanjing 210096, China; Plastic Surgery Hospital, Chinese Academy of Medical Sciences and Peking Union Medical College, Beijing 100043, China; State Key Laboratory of Bioelectronics, School of Biological Science and Medical Engineering, Southeast University, Nanjing 210096, China; Avatarget Co. Laboratory of Organs-on-a-Chip Research, Suzhou 215163, China; State Key Laboratory of Bioelectronics, School of Biological Science and Medical Engineering, Southeast University, Nanjing 210096, China; Avatarget Co. Laboratory of Organs-on-a-Chip Research, Suzhou 215163, China; State Key Laboratory of Bioelectronics, School of Biological Science and Medical Engineering, Southeast University, Nanjing 210096, China; Avatarget Co. Laboratory of Organs-on-a-Chip Research, Suzhou 215163, China; Plastic Surgery Hospital, Chinese Academy of Medical Sciences and Peking Union Medical College, Beijing 100043, China; State Key Laboratory of Bioelectronics, School of Biological Science and Medical Engineering, Southeast University, Nanjing 210096, China; State Key Laboratory of Bioelectronics, School of Biological Science and Medical Engineering, Southeast University, Nanjing 210096, China; Plastic Surgery Hospital, Chinese Academy of Medical Sciences and Peking Union Medical College, Beijing 100043, China

**Keywords:** skeletal muscle, microenvironment, microphysiological system, *in vitro* modeling, bioengineering

## Abstract

Research on myogenesis and myogenic pathologies has garnered significant attention in recent years. However, traditional *in vitro* modeling approaches have struggled to fully replicate the complex functions of skeletal muscle. This limitation is primarily due to the insufficient reconstruction of the muscle tissue microenvironment and the role of physical cues in regulating muscle cell activity. Recent studies have highlighted the importance of the microenvironment, which includes cells, extracellular matrix (ECM) and cytokines, in influencing myogenesis, regeneration and inflammation. This review focuses on advances in skeletal muscle construction toward a complete microphysiological system, such as organoids and muscle-on-a-chip technology, as well as innovative interventions like bioprinting and electrical stimulation. These advancements have enabled researchers to restore functional skeletal muscle tissue, bringing us closer to achieving a fully functional microphysiological system. Compared to traditional models, these systems allow for the collection of more comprehensive data, providing insights across multiple scales. Researchers can now study skeletal muscle and disease models *in vitro* with increased precision, enabling more advanced research into the physiological and biochemical cues affecting skeletal muscle activity. With these advancements, new applications are emerging, including drug screening, disease modeling and the development of artificial tissues. Progression in this field holds great promise for advancing our understanding of skeletal muscle function and its associated pathologies, offering potential therapeutic solutions for a variety of muscle-related diseases.

## Graphical abstract

**Figure:**
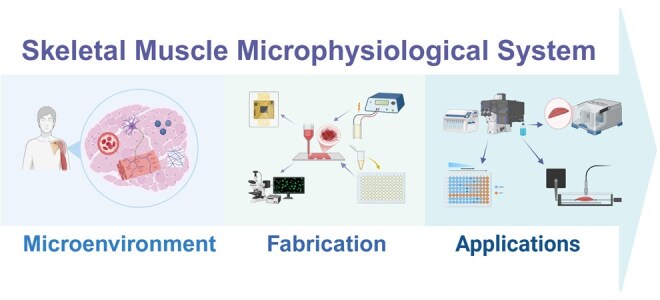

## Introduction

**Figure 1.**
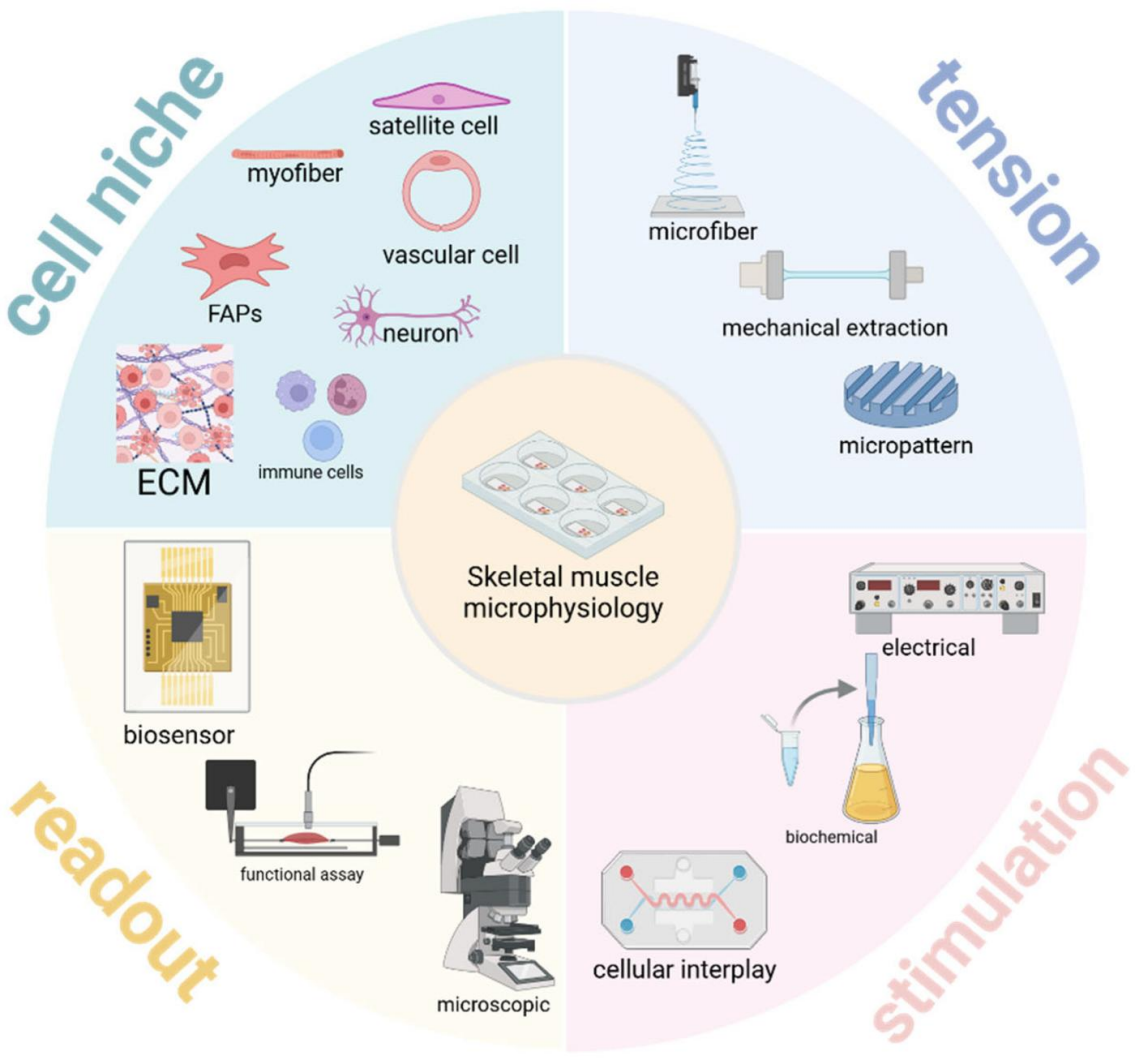
Engineering of skeletal muscle tissue in microphysiological system. Created in BioRender. gao, y. (2025).

Skeletal muscle, one of the largest organs in the human body, is widely distributed and plays a crucial role in physiological processes as well as in pathological changes caused by diseases, injuries, aging and other potentially impactful conditions. Experiments involving animals and humans in skeletal muscle research are limited in their application. The morphology, size and structure of animal muscles differ significantly from those of the human body, reducing the applicability of experimental findings. Additionally, human experiments are ethically constrained due to the trauma and functional impact on the donor muscle. In summary, more sophisticated *in vitro* models are required, as traditional models have failed to meet research needs, driving the development of new models. The growth, development and function of skeletal muscle depend on various physiological and biochemical factors. The construction of *in vitro* models necessitates the incorporation of numerous elements, involving multidisciplinary engineering strategies beyond the scope of traditional models, thereby requiring the establishment of a microphysiological system to accurately mimic human skeletal muscle (shown in Figure 1). This article reviews the composition of the skeletal muscle microenvironment, as well as current advancements in microphysiological systems for simulating skeletal muscle, and their applications and future directions in research.

## Microenvironment of skeletal muscle tissue

**Figure 2.**
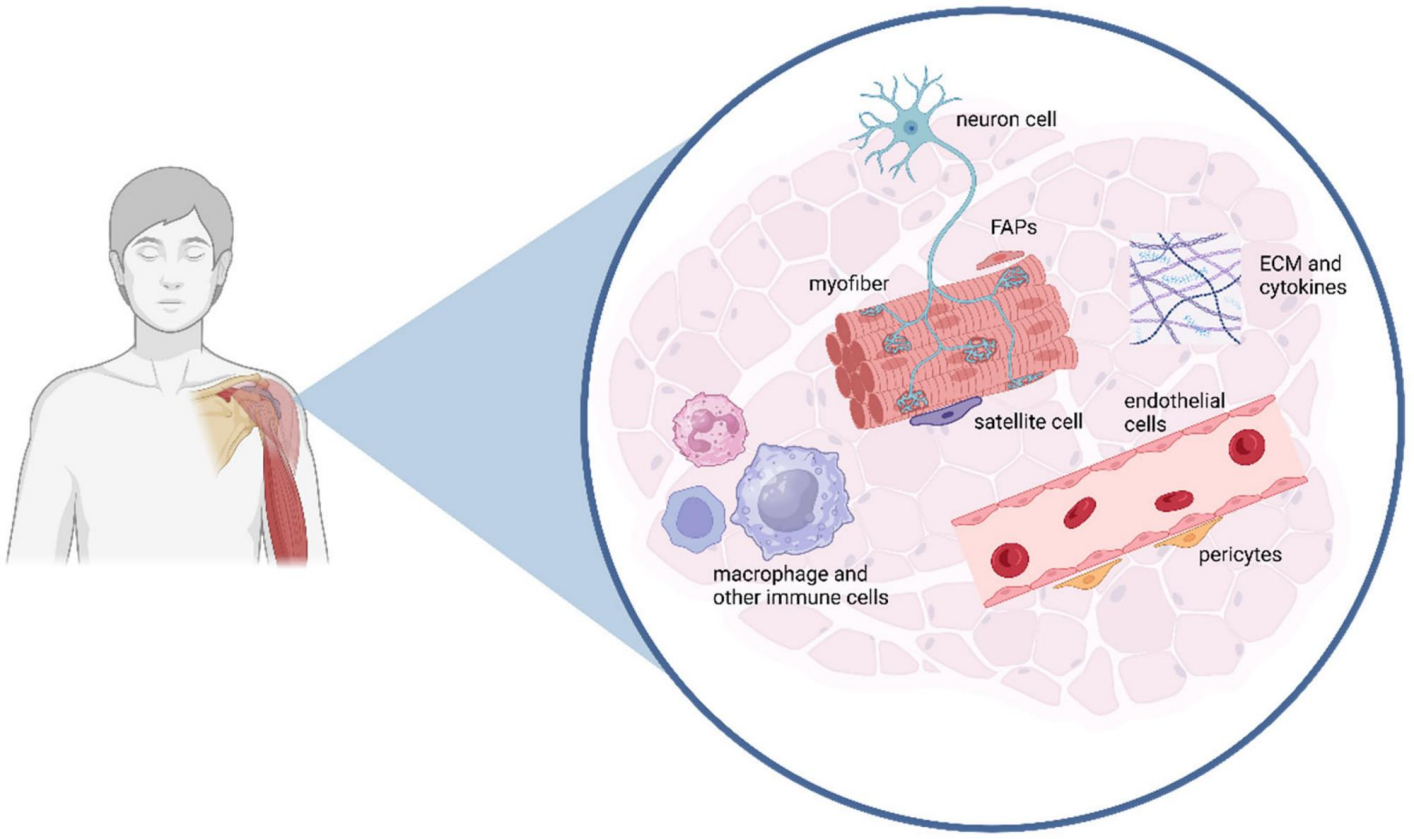
The microenvironment of skeletal muscle. Created in BioRender. gao, y. (2025). The microenvironment of skeletal muscle includes muscle progenitor cells like satellite cells, extracellular matrix, blood vessels, immune cells and other components. A stable cellular microenvironment ensures normal myocyte function, maintains satellite cells quiescent and is jointly regulated by internal and external factors.

Skeletal muscle tissue comprises a highly organized arrangement of muscle fibers encased within an extracellular matrix (ECM) that includes collagen, elastin, glycoproteins, proteoglycans and other proteins [1] (as shown in Figure 2). The ECM contains an extensive network of capillaries and nerves and hosts a variety of cells, including muscle stem cells (including satellite cells (SCs)), fibroblasts, immune cells and fibro-adipogenic progenitor cells (FAPs) [2]. Together, these components constitute the microenvironment essential for myoblast growth. A variety of physiological processes and functions, including myoblast development and injury repair, rely on the mediation and support of an intact microenvironment.

### Progenitor cells

SCs are a heterogeneous population of muscle stem cells located primarily beneath the basal lamina and adjacent to the basement membrane. Pax7 is recognized as the characteristic protein expressed by SCs. SCs typically remain quiescent but are activated in response to muscle injury. The cell fate of SCs involves both self-renewal and differentiation, regulated by intrinsic factors (e.g. cell cycle regulators) and extrinsic factors (e.g. ECM and other cells) [3]. Quiescent SCs are activated and undergo several cycles of proliferation in response to muscle damage or stress. Proliferating SCs may differentiate into myoblasts or return to a quiescent state to replenish the SC pool [4]. Maintaining SC and muscle homeostasis over time requires a balanced transition between quiescence and activation [5].

Fibro-adipogenic progenitors (FAPs) are mesenchymal progenitor cells residing in the mesenchymal region of skeletal muscle, playing a pivotal role in long-term maintenance, regeneration and growth of muscle [6]. In the resting state, FAPs remain quiescent but become rapidly activated and proliferate following acute injury, peaking in number 72–96 h post-injury. FAP activation leads to a transient increase in ECM production [7]. Under normal conditions, FAP proliferation and differentiation are restricted; however, abnormalities can lead to fibrosis and steatosis in muscle tissue. IL-15 promotes FAP proliferation via the JAK-STAT pathway and inhibits adipogenesis through the DHH pathway [8]. The miR-22-3p/KLF6/MMP14 signaling axis regulates FAP differentiation [9]; animal experiments have demonstrated that the TGF-β/BMP pathway has a regulatory function in the FAPs [10]. The major downstream effector and the enzyme responsible for TGFβ/BMP gene expression in FAPs is matrix metalloproteinase-13 (MMP-13), whose gene ablation suppresses TGFβ/BMP signaling in FAP fibro/adipogenesis [10]. The majority of the FAP subpopulation expresses TEK/TIE2 receptor tyrosine kinase (TIE2) during the resting state [11].

### Extracellular matrix

Myofibers are surrounded by ECM composed of collagen, various glycoproteins such as fibronectin and laminin, matrix metalloproteinases (MMPs) and matricellular proteins [12]. Connection and communication between myocytes and the surrounding matrix guarantee the basic functions of the myofiber.

Collagen presents a function to inhibit differentiation and quiescent SCs. The exact mechanism of this process in type I collagen remains unknown, whereas type V may act through the collagen–notch–calcitonin pathway [13]. Type VI is a major component of the muscle ECM and plays a role in maintaining a pool of SCs, although the exact mechanism remains to be investigated [14].

Fibronectin is crucial for maintaining the structure and function of tendon junctions, regulating muscle integrity and facilitating vesicular transport of myotonic dystrophy proteins [15]. Highly expressed in the ECM of mature differentiated skeletal muscle, fibronectin mediates mitosis and expansion of SCs [16]. Intracellular nuclear motility, ECM secretion and SC fate regulation through integrin adhesion to myofibroblasts are also regulated by fibronectin [17, 18].

Laminin is involved in the formation of the basement membrane. A lack of Laminin-211 aberrantly activates the JAK-STAT pathway, leading to enhanced asymmetric division of SCs, damaging them and impairing muscle repair and normal development [19]. Laminin-111 reduces pathology in animal models, maintains SC polarity and regulates asymmetric cell division [20].

The first MMP was discovered in 1962 in the tail of a tadpole and was shown to degrade collagen. As the name implies, MMPs are proteases that remodel the ECM. MMPs have been shown to play many additional biological roles in cellular interplay, immunoregulation and transcriptional regulation [21]. As previously mentioned, MMP13 and MMP14 are involved in regulating muscle adipogenesis and fibrosis.

Given these critical functions, ECM plays a significant role in the bioactivity of muscle cells. Researchers have brought decellularized ECM into tissue engineering in order to bring in necessary biochemical cues and acquire more functional tissue models [22–25].

### Immune cells

Macrophages within muscle tissue exhibit unique heterogeneity [26]. Following muscle injury, monocytes from the circulatory system are recruited into muscle tissue, guided by CCR2 signaling. Monocytes are recruited to the injured site, differentiate into pro-inflammatory macrophages and trigger a series of actions. Macrophages are responsible for phagocytosing cellular debris and secreting pro-inflammatory cytokines during the inflammatory phase. These cytokines promote muscle stem cell proliferation and initiate the activation of endothelial cells (ECs) and FAPs. Later, the pro-inflammatory phenotype shifts to an anti-inflammatory phenotype. By resolving minor lesions to curb inflammation and eliminating muscle tissue debris after injury, macrophages help preserve homeostasis [2, 27].

T cells directly enhance myogenesis by facilitating the migration and proliferation of muscle SCs *in vitro*, primarily through the secretion of IL-1α, TNF-α, IFN-γ and IL-13 [28].

### Vascular system

Skeletal muscle contains the highest density of microvessels and dynamically adapts to environmental and physiological changes. ECs in healthy muscle act as metabolic sensors and are key determinants of muscle oxygen uptake [29]. Pericytes are mural cells surrounding ECs in capillaries, precapillary arterioles and postcapillary venules. They influence angiogenesis by promoting the migration, proliferation and differentiation of vascular ECs [30]. In addition to these functions, pericytes exhibit stemness similar to mesenchymal stem cells (MSCs) [31]. Pericytes can be identified by the expression of growth factor receptor PDGFRb and proteoglycan NG2, a co-receptor of PDGF [30].

### Muscle stem cell communication with other cells

#### Macrophages and muscle stem cells

Macrophages regulate SCs through paracrine and direct contact [32, 33]. Studies in skeletal muscle tissue engineering have demonstrated that macrophages can repair muscle tissue with impaired regenerative function [34] and that adult myoblasts secrete cytokines and chemokines to regulate immune cell function [35]. Accordingly, Pax7+ SCs influence macrophage phenotypic transformation. Even with pro-regenerative cytokine treatment, cardiotoxin (CTX)-induced damage reduced the SC pool in a murine model of myogenic cell-engineered skeletal muscle tissue, leading to progressive tissue degradation. Nevertheless, adding bone marrow-derived macrophages to engineered tissues promotes SC differentiation and proliferation, leading to nearly complete muscle restoration and highlighting the significance of intercellular connections between SCs and macrophages in this process [34].

#### FAPs and muscle stem cells

FAPs located in the vicinity of injured muscle fibers support the proliferation of myoblasts and transform the surrounding microenvironment into a state that promotes differentiation [36]. FAPs’ expansion following injury is correlated with their interactions with other muscle cell populations. Myofibrils control FAP differentiation through pro-fibrogenic and anti-adipogenic factors, while soluble factors from myofiber progenitors activate the PI3K/AKT pathway, promoting proliferation [4]. In DMD disease models, the interaction is disrupted and the number of FAPs is upregulated, causing muscle fibrosis, adiposity, impaired muscle tissue healing and aggravation of degeneration [37, 38]. Dynamic TGF-β1-mediated communication between FAPs and macrophages allows FAP to regulate macrophage polarization, partly through Osr1 expression. FAPs also influence SC destiny by interacting with macrophages [39].

#### ECs and muscle stem cells

During regeneration, SCs can induce the proliferation and migration of ECs. *In vitro* co-culture experiments have demonstrated that ECs promote myogenic cell growth by secreting soluble factors, including IGF-1, hepatocyte growth factor (HGF), basic fibroblast growth factor (bFGF), PDGF-BB and VEGF [40]. Moreover, ECs secrete apelin, oncostatin M and osteopontin, which coordinate the myogenesis/angiogenesis coupling during repair and stimulate the migration, proliferation and differentiation of myogenic precursor cells [41]. Muscle stem cells and differentiated myogenic cells both secrete substances that promote angiogenesis and sustain the regeneration of VEGF [40], β-catenin and hypoxia-inducible factors [2]. Similarly, endothelial progenitor cells are recruited to the site of muscle injury to promote the development and migration of myogenic cells [41].

### Skeletal muscle and mechanosignaling

Motor neurons (MNs) and muscle fibers interact during skeletal muscle development to form neuromuscular junctions (NMJs). The NMJ is a specialized chemical synapse consisting of a presynaptic membrane, a postsynaptic membrane and a synaptic cleft, serving as the site where neuron axons terminate on skeletal muscle fibers. Each MN sends long axons, or nerve fibers, to the muscles it innervates. Near the muscle cell, each nerve fiber branches into tens or hundreds of extensions. These extensions often form a one-to-one NMJ with individual muscle fibers. Voltage-gated calcium channels in the presynaptic membrane open in response to an action potential reaching the axon terminal. Acetylcholine (ACh), one of the most common neurotransmitters, is released when calcium ions enter and cause synaptic vesicles within the cell to fuse with the presynaptic membrane [42]. After being released, ACh diffuses across the synaptic cleft and binds to the ACh receptor (AChR) on the postsynaptic membrane of muscle fibers. This binding activates sodium channels in the sarcolemma, and the resulting ion flow triggers an action potential that propagates to the sarcoplasmic reticulum. Muscle contraction results from the release of calcium ions into the cytoplasm from the sarcoplasmic reticulum. Acetylcholinesterase, located in the synaptic cleft of the muscle fiber's basement membrane, rapidly deactivates ACh in the synaptic cleft and terminates muscle excitation [42].

Integrin receptors and mechanosensitive ion channels, such as transient receptor protein ion channels and piezoelectric ion channels, are able to sense mechanical strain, which can be conveyed across the ECM and adjacent cells. Adhesion patch kinase (FAK) and several other signaling pathways, such as RhoA/Rock, Wnt/β-catenin, MAPK, TGF-β and Pi3K pathways, are then activated as a result. These pathways control the expression of many myogenic genes as well as other cellular responses, including cell division and cytoskeleton structure [43–45].

## *In vitro* models of skeletal muscle organization

### Planar cell culture model

**Figure 3.**
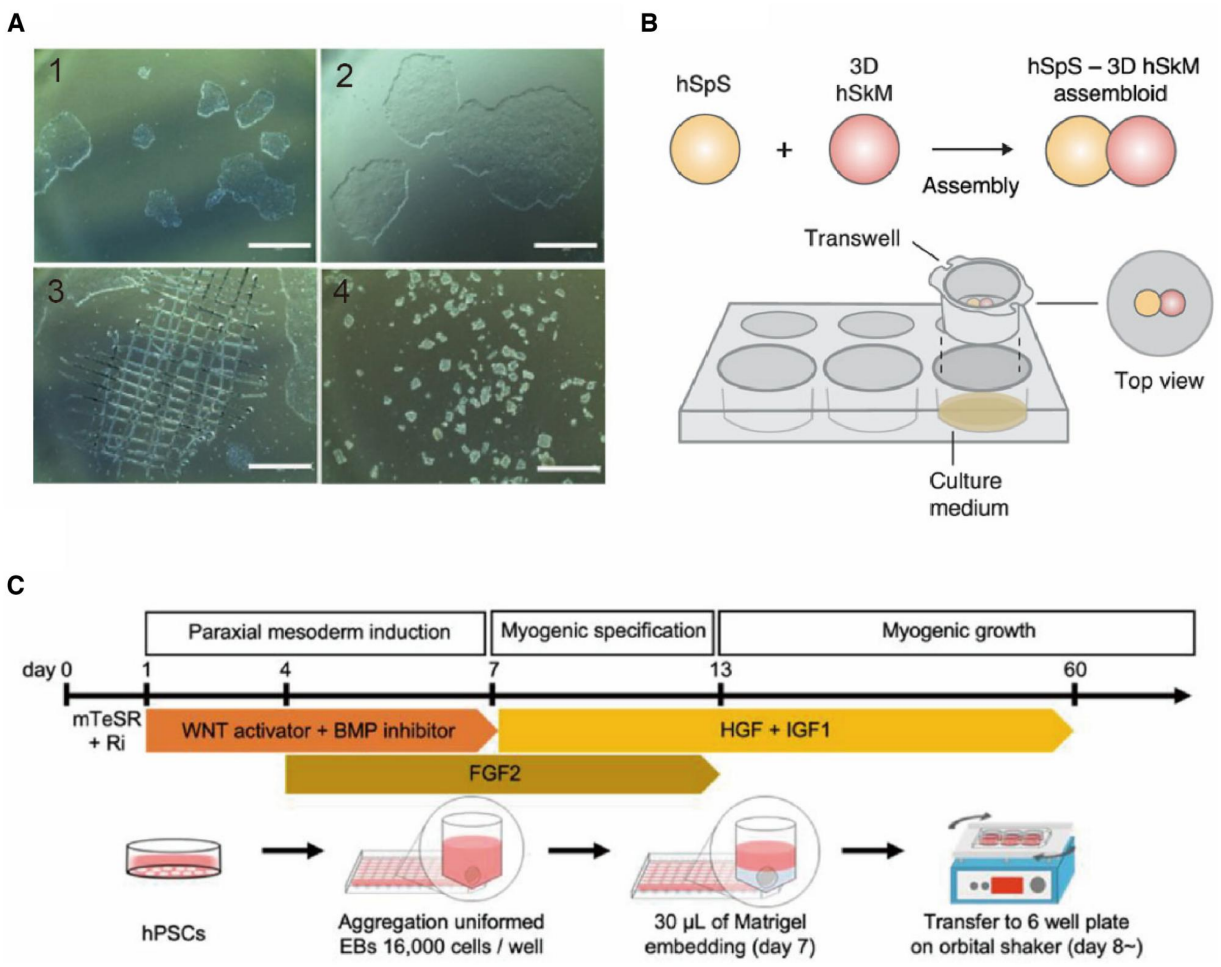
Planar culture and organoid models of skeletal muscle. (**A**) Myoblast colonies differentiated from hPSCs in a planar culture system over different time points during the process, the number in each section shows the time order [46]. (**B**) Schematic of co-culture system for human motor neurons and skeletal muscle cells in a Transwell model for neuromuscular junction formation. hSpS: human spinal cord cells, hSkM: human skeletal muscle cells [47]. (**C**) A protocol scheme for inducing hPSC differentiation into myogenic progenitor cells using small molecules, divided into three distinct phases: paraxial mesoderm induction, myogenic induction and myogenic growth [48].

Culturing on planar substrates is the most straightforward, simple and cost-effective technique for cytological studies. Most early attempts at constructing *in vitro* skeletal muscle models were initially based on planar culture systems [49–51]. The planar model offers advantages of simplicity and ease of operation; however, conventional culture media cannot adequately replicate the 3D structure of the tissue microenvironment or restore the functional units at the periphery, presenting a limitation. For example, aligned myotube formation has been proved to require guidance like patterning, which is unavailable in a traditional culture model [52]; the 3D relationship between fibroblasts and myocytes [53] cannot be fully replicated on a 2D plate. As 3D culture technologies have advanced, attempts to use planar culture for myotubular differentiation have become increasingly rare in recent years [54, 55]. During the transformation of pluripotent stem cells into myofibroblasts, many researchers have opted for 2D culture systems [56–58] (as shown in Figure 3).

### Organoids

Planar culture cannot fully replicate the complex functional units and microenvironment of human muscle tissue; therefore, researchers have introduced 3D culture techniques. Organoids are constructed based on this technique, wherein differentiated cultures self-assemble through the differential adhesion of cells from different tissues, recapitulating the muscle fiber structure and microscopic functional units such as neuromuscular connections, microvessels, ECM and other components [59] (as shown in Figure 3). Mavrommatis *et al.* [60] and Shin *et al.* [48] induced the differentiation of human iPSCs using a small molecule combination to form muscle organoids, mimicking fetal myofibrillogenesis via this model. Martins *et al.* [61] used hPSC-derived axial stem cells to generate both spinal cord neurons and skeletal muscle cells, which self-organized into human neuromuscular organoids containing fully functional neuromuscular connections. Efforts toward microvascularization of muscle organoids have been ongoing to preserve their function over time. Researchers like Minne *et al.* [62] have worked to prolong the survival of muscle organoids by introducing ECs, thereby enhancing their utility in applications such as drug screening.

### Microphysiological systems

The primary function of muscle tissue in the human body is to facilitate mechanical movement, making tension a critical factor in the physiological environment of skeletal muscle and a key signal for muscle growth and differentiation [63, 64]. However, organoid culture techniques are unable to replicate this physiological aspect of muscle fascicle generation. Current methods for measuring contractile force rely on assessments of elastic modulation and resulting movement, necessitating uniaxial and directed force application; however, spheroids do not fulfill this requirement. Self-assembled organoids are also limited in generating associated tissues, such as tendons, which restricts their ability to simulate functionality [65]. Additionally, early culture methods constrain the survival time of muscle tissue in *in vitro* models. As methods for differentiating and growing myoblasts into myotubular tissues improved, researchers began constructing skeletal muscle microphysiological systems to cultivate functional myoblast tissues.

### Involvement of key factors in the skeletal muscle microphysiological system

*In vitro* models aim to replicate the growth conditions of human skeletal muscle tissue. To achieve this, constructing a skeletal muscle microphysiological system requires integrating various physiological and biochemical factors to simulate the human microenvironment and produce functional muscle tissue. Given the extensive motor and electrophysiological activities inherent in skeletal muscle, the assessment of these tissues cannot be confined to traditional histological and molecular tests. Instead, there is a need to incorporate multidimensional indicators and advanced measurement techniques. As such, the integration of key factors and the acquisition of physiological and biochemical signals through bioengineering techniques are critical technical priorities in current research. The microphysiological system enables the introduction of force, electrical stimulation and various other biophysical factors into the microenvironment (see Table 1). Coupled with the presence of signal pathways such as PIEZO1, as previously mentioned, bioactivity can achieve a higher degree of simulation of actual cell activities. By integrating with specially designed machines and instruments, we can obtain controllable and quantified input of these factors, thereby enhancing the reproducibility and reliability of the engineered tissue (see Table 2).

**Table 1.** Comparison of different skeletal muscle models

	Tissue functionality	Physiological Relevance	Scalability	Feasibility		
				Reproducibility	Availability	Cost
Planar model	Limited cell types: muscle cells, myotubes and mesenchymal cells	Cellular activities and molecular mechanisms.	Low: Short-term culture, cell morphology observation, and cell-level intervention.	low	high	low
Organoids	Multiple cell types and functional units: neuromuscular junctions, vascularized organoids, iPSC-derived spheroids	Cellular interplay,3D microenvironment, tissue development(e.g. myogenesis).	Medium: Long-term culture, observation of differentiation and development over time, and intervention based on cellular crosstalk.	low	medium	high
Microphysiological system	Various cell types with aligned architecture and a defined microenvironment: functional myobundles, neuromuscular junctions, blood vessels, oxygen uptake systems, immune microenvironment and extracellular matrix (ECM)	Interplay at level of cell-to-cell, cell-to-microenvironment and tissue-to-tissue.	High: Long-term culture with on-chip in situ tissue morphology observation, mechanical testing, and electrophysiological analysis.	high	low	high

**Table 2.** Comprehensive review of current microphysiological system construction

Strategy	Material and scaffold	Cell type	Model design	Intervention
Fulcrum	Fibrin and Matrigel/Geltrex	C2C12 [66]	Laser-cut Cerex frames were placed in polydimethylsiloxane (PDMS) molds containing two semicylindrical wells	Vascularization Dynamic culture Electric pulse stimulation
		Primary human myoblast [67, 68]		
		IPSC-derived myoblast [57]		
		IPSC-derived myoblast	Two pillars surrounded by a cell-hydrogel mixture [55]	Neural and epithelial cells co-culture
		Primary human myoblast	A 96-well size chamber with two pillars and hooks on its top [69]	Chemical and electrical stimulation
		iPSC-derived myoblast	Chamber with two pillars [70]	Electrical stimulation
		Primary human myoblast	Chamber with two electrodes as fulcrum [71]	Electrical pulse stimulation
		Human primary myoblast	One-chamber with two pillars [72]	Electrical and chemical stimulation Co-culture with neurons
	GeIMA	C2C12 [73]	Two pillars in a chamber with a microfluidic device. [73]	Mechanical tension
	Growth factor reduced Matrigel	C2C12	PDMS film with stretcher [74]	Mechanical tension
	Collagen I and Matrigel	IPSC-derived myoblast	A two-chamber microfluidic device with two pillars in the motor unit [75, 76]	Electrical and chemical stimulation Co-culture with neurons
		IPSC-derived myoblast	Growth on a single pillar and maturation with isometric load on two hooks [59]	Electrical and mechanical stimulation
		Primary human myoblast	One-chamber microfluidic chip with two pillars and platinum wires as electrodes [77]	Electrical pulse stimulation
	Xeno-free hydrogel with human platelet lysate	Human immortalized muscle satellite stem cells	Chamber with two pillars [78]	Electrical pulse stimulation
Patterning	GeIMA	C2C12	Patterned surface with acoustic wave [79]	–
	Matrigel	IPSC-derived myoblast	PDMS mold replicated by photolithography-fabricated silicon wafer [80]	–
	GelMA-carboxymethyl cellulose methacrylate (CMCMA)	Immortalized human myoblasts	PDMS mold replicated by photolithography-fabricated silicon wafer [81]	–
	Gelatin	HPSC-derived myoblast	PDMS mold replicated by laser-engraved glasslips [82]	Electrical stimulation
Bioprinting and microfiber	Alginate/fibrin fiber	Human-derived adipose-derived stem/stromal cells	Electrospinning [83]	Plant *in vivo*
		C2C12/3T3	Electrospinning [56]	–
		hESC-derived myoblast	Electrospinning [84]	Plant *in vivo*
	Hyaluronic acid (HA), gelatin, and fibrinogen	C2C12	Bioprinting into different shapes [85]	Electrical stimulation
	HA, gelatin, fibrinogen and glycerol	Primary human myoblast	Three-dimensional bioprinting [86]	Plant *in vivo*
	Collagen I	C2C12	Hybrid biprinting [87]	Plant *in vivo*

## Physiological and biochemical factors to be involved

### Tension involvement

**Figure 4.**
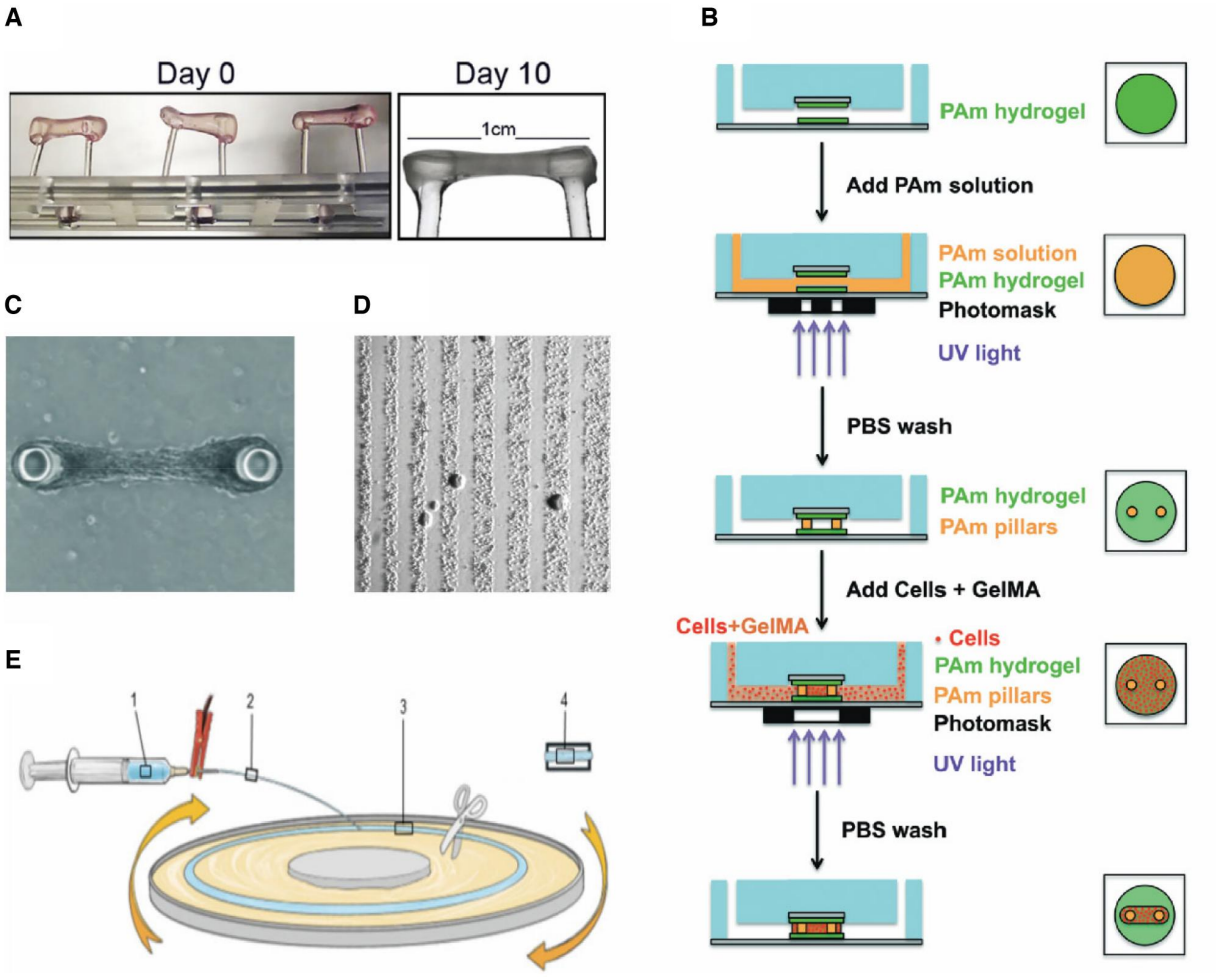
Different attempts to introduce tension into muscle microphysiological systems. (**A**) Schematic diagram of applying tension to myoblasts encapsulated in a hydrogel to induce myofascial generation [55]. (**B**) Process of fabrication of a muscle-on-chip device with two pillars set up to provide fulcrums for myogenic cells, which utilize various photo-curable materials for model fabrication. PAm: acrylamide (Am) solution containing photoinitiator, GelMA: gelatin methacrylate [73]. (**C**) Microscopic images of the muscle-on-a-chip device after culture maturation, showing a bundle-like structure [73]. (**D**) Directing myofibrillar generation by sculpting surface patterns to provide surface tension to myocytes [84]. (**E**) Schematic of microfiber production for muscle cell alignment, involving cell and hydrogel mixture loading into a syringe, extrusion under applied voltage, fiber formation on a rotating platform and final collection [80].

Tension is a persistent physical factor essential for muscle growth and development, playing a crucial role in the orderly formation of myotubes [52]. Mechanosignaling pathways, such as PIEZO1, directly mediate the growth and development of myocytes. The establishment of an intact and organized myofiber structure, which is fundamental to muscle function, is dependent on the presence of tension. Additionally, tension significantly influences various physiological processes, including muscle inflammation, damage repair and regeneration [64]. Current approaches to introducing tension into *in vitro* culture systems include the use of fulcrums, surface tension guidance and artificial fiber scaffolds (shown in Figure 4).

#### Provide fulcrum

When myogenic cells are inoculated into a 3D medium for differentiation and provided with fulcrums, the differentiated myotubes and the surrounding medium spontaneously contract and reorganize, forming a muscle bundle-like structure that attaches to the fulcrums at both ends [72, 73, 83, 88]. Using the fulcrum, the myotube can be stretched, and the applied tension quantified [74, 89, 90]. This strategy minimally disrupts myotube differentiation and facilitates myobundle formation, with adjustable variables primarily lying in the design of the fulcrums. Agrawal *et al.* [73] examined myofiber production under varying fulcrum diameters and spacing conditions, finding that myofibers grew optimally at a specific ratio of fulcrum spacing. Additionally, Afshar *et al.* [72] reported that hooked fulcrum ends had a higher success rate of myofibril attachment.

#### Surface tension

Micropatterning has been a classical strategy to build up proper cell alignment [52, 91, 92]. By providing different substrate patterns and stiffnesses, myocytes are allowed to obtain surface tension from the culture plane to guide their growth by different materials [52, 80], photoplastic engraving [93], mechanical vibration [79], etc. In a study by Jiwlawat *et al.* [80], it was found that culturing myotubes on a soft substrate, such as hydrogel, with parallel textures engraved on the surface induced myotube growth in an orderly fashion, forming parallel structures. Afshar *et al.* [69] demonstrated that models with hooked pivot ends exhibited a higher success rate of muscle bundle attachment. In addition to surface texture, some researchers have created micropores and threads within the culture medium, directing cell growth along the engineered paths to form artificial muscle textures. The advantage of such microstructural designs is that they can simulate the anisotropy of muscle fibers and the intersection of muscle force direction at a 3D level [94–96], a critical factor in the 3D shaping of muscle contours in the human body [97]. This engineering strategy has enabled researchers to intervene more effectively in the direction of skeletal muscle growth and formation within microphysiological systems.

#### Artificial fibers and bioprinting

In addition to providing surface tension, another engineering strategy to artificially intervene in the direction of myotube growth is bioprinting. A typical solution involves modifying the hydrogel medium into a fibrous morphology through electrospinning and mechanical stretching, followed by inoculation with myocytes, guiding tissue formation along the direction of the microfibers [83, 84, 87, 98–101]. This engineering strategy provides a more refined approach for influencing the structure of skeletal muscle tissues. Volpi and Kiratitanaporn [56, 102], in their study, mimicked the fiber arrangement in tendon tissues by printing fibers to guide fibroblast arrangement, addressing the issue of disordered fibroblast organization in natural cultures. Additionally, Kim *et al.* [86] used bioprinting to integrate neural cells into artificial skeletal muscle tissues. Zhang *et al.* [100] took advantage of this strategy and managed to replicate the anisotropy in their study.

### Electrical and chemical stimulation involvement

**Figure 5.**
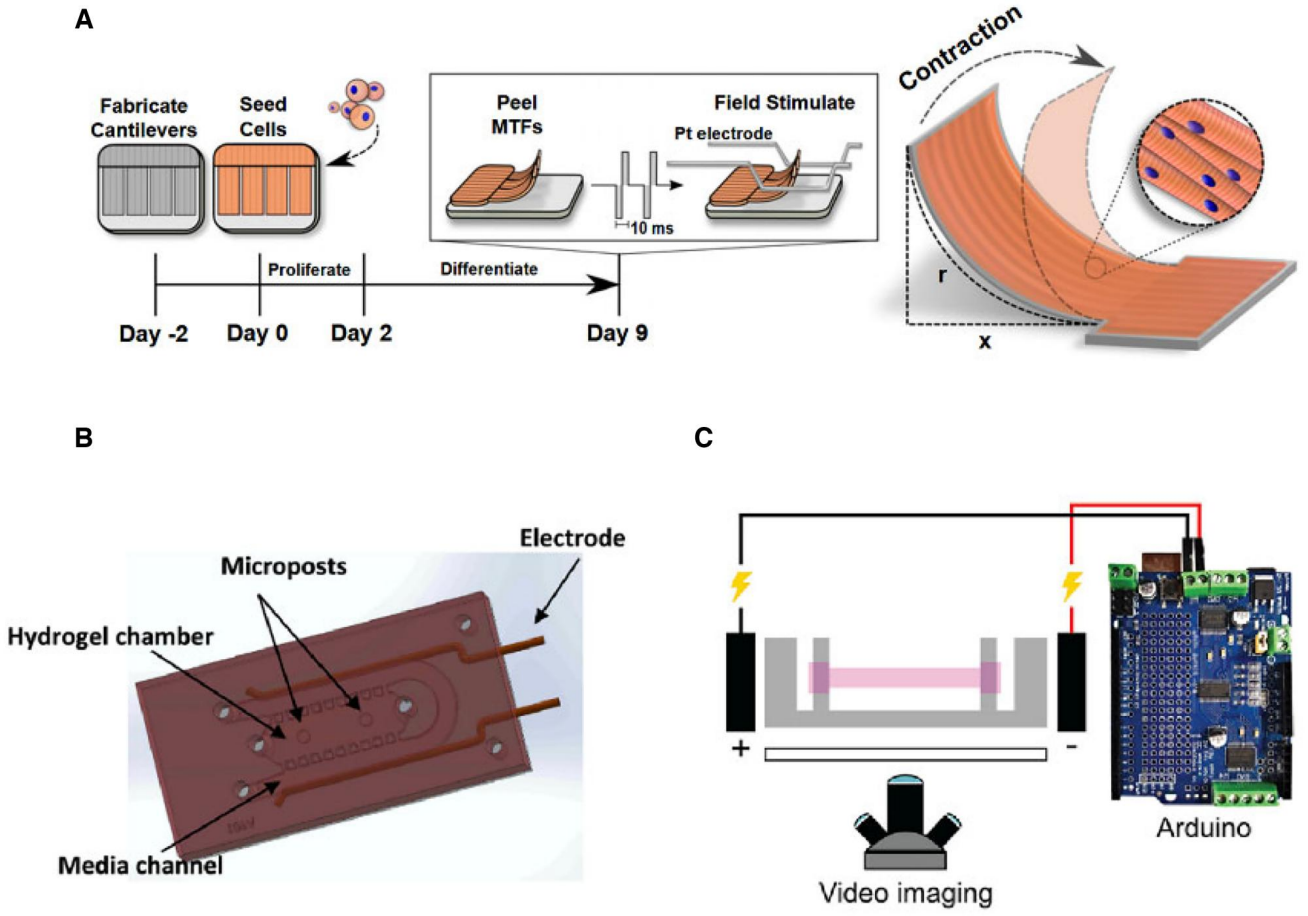
Introduction of electrical stimulation and activation of muscle to the skeletal muscle microphysiological system. (**A**) Cultured muscle tissue was placed into an electric field to induce muscle contraction [82]. (**B**) Electrodes were introduced into the microfluidic chip to create organoids to simulate muscle contraction [77]. (**C**) Electrodes were inserted into the cultured complete muscle bundles, and the cyclic electrical stimulation inputs were fed through the artificial circuitry to emulate the muscle physiological environment [70].

Stimulated contraction is a fundamental manifestation of muscle function, and the most straightforward verification involves applying electrical stimulation to induce muscle contraction [71, 86, 103] (as shown in Figure 5). In Pallotta’s study [71], muscle tissue responded to electrical stimulation at frequencies ranging from 1 to 100 Hz, generating tetanic forces between 0.09 and 0.55 mN/mm^2^. Similar increases have also been observed by Khodabukus *et al.* [103] and other researchers in their research. This pattern closely resembles physiological conditions. Additionally, electrically induced contractions trigger physiological and biochemical changes in myocytes.

Consequently, introducing electrical stimulation systems into microphysiological systems has become a prevalent engineering strategy for developing functional tissue models. Methods for applying electrical stimulation include integrating electrodes or situating muscle tissues in electric fields for stimulation. Researchers can adjust various parameters to tailor experimental triggers [71, 104]: different intensities of electrical stimulation can induce muscle contractions or spasms, while modulating the electric field frequency can generate stimulation at varying frequencies and intensities, mimicking different physiological conditions of muscle function [71, 105].

The presence of NMJ in functionally intact muscle tissue allows the addition of ACh to the culture system to activate AchR and induce contraction, similar to electrical stimulation applied to the neural component of a musculoskeletal co-culture system [95]. Additionally, chemicals such as thyroxine, caffeine and epinephrine [59, 106] enhance muscle contraction by acting on their corresponding receptors. AchR inhibitors, including tetrodotoxin [77] and cylindrospermopsin [67], suppress receptor activity and muscle function.

### Mechanical and chemical injuries involvement

**Figure 6.**
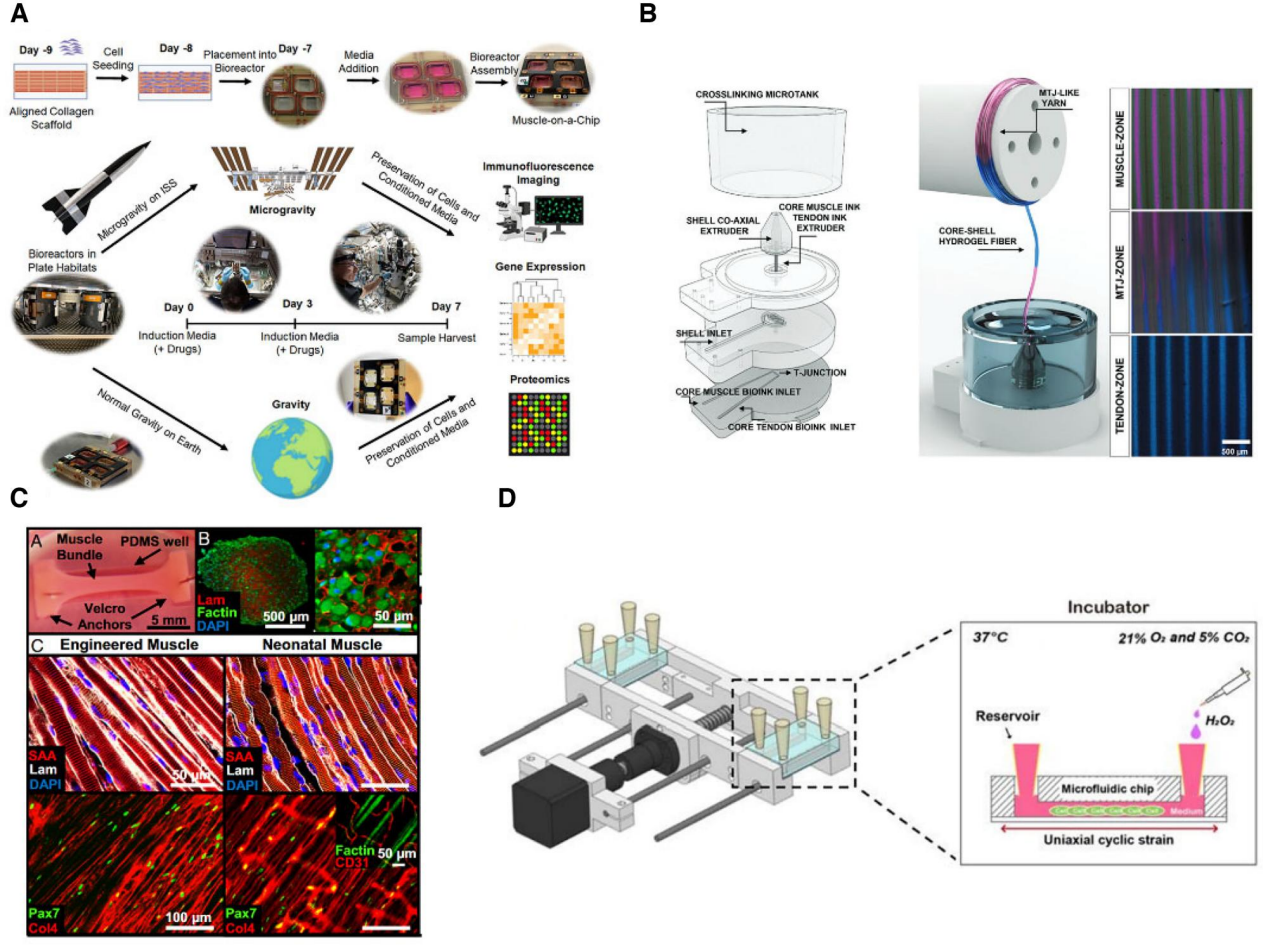
Introduction of other physiological and biochemical factors into skeletal muscle microphysiological systems. (**A**) Fabrication of organoids by microfiber-guided muscle generation and sending them to microgravity environments for comparative studies in culture, comparing functional and pharmacological validation [99]. (**B**) Obtaining musculotendinous junction tissues by hybrid 3D printing [56]. (**C**) Introduction of endothelial cells for the cultivation of vascularized artificial muscle bundles [107]. (**D**) Introduction of mechanically and chemically damaged microphysiological systems [74].

Muscle lesions include both physical and chemical injuries, and the application of overloaded tension can simulate mechanical damage caused by external forces, such as muscle tearing [74]. For toxic damage to muscle, a common modeling option is CTX [59, 73, 107], a toxin found in snake venom that promotes myotube depolarization and disrupts the muscle cytoskeleton. It is frequently used in experiments to induce skeletal muscle damage in animal models (as shown in Figure 6). Other alternatives include H_2_O_2_ and various cytotoxic chemotherapeutic agents [88, 107].

### Microgravity involvement

Muscle dysfunction under weightlessness or microgravity has been a focus of study since the space age began. Kim and colleagues [99] deployed a microphysiological system in the space microgravity environment, which can simulate muscle disuse to some extent (shown in Figure 6). For researchers without access to spacecraft, the random position machine (RPM) offers a viable alternative for simulating microgravity [108]. When combined with microfluidic systems, *in vitro* cultures under microgravity reduce manual handling, improve experimental continuity and better mimic physiological conditions.

### Signals by tissue-cross talk

Skeletal muscle interacts with neural tissues [109, 110] and tendon tissues [56, 102, 111] and mechanistically links to them. Together with the organ-on-a-chip technology, multiple tissues can be co-cultured, and through appropriate model design, independent tissue units can be established, their interactions can be designed and functional units such as NMJ and muscle–tendon junctions can be simulated for study (shown in Figure 6). In a successfully constructed neuromuscular microphysiological system, electrical stimulation of the nerve side can cause contraction of the muscle side, visually demonstrating the integral nature of the neuromuscular connection and the muscle's own function [95].

### Biological factors and extracellular environment

Myocytes interact extensively with the ECM under physiological conditions, as well as with various biological factors in their environment, which collectively help maintain muscle homeostasis. As a crucial component of tissue engineering, the selection of scaffold materials plays a significant role in the development of engineered muscle models. These scaffolds can be derived from either natural or synthetic materials. Decellularized ECM (dECM) is a classic natural biomaterial, offering a comprehensive biological composition that closely mimics native muscle tissue [112]. In contrast, hydrogels are among the most commonly used synthetic materials, with polymer-based hydrogels being widely applied due to their structural similarity to the ECM of various tissues and organs [113]. These hydrogels provide critical structural support and help regulate the cellular microenvironment. Among them, fibrin-based and collagen-based hydrogels are the most widely utilized and have been shown to effectively support muscle cell differentiation in research studies [114, 115]. The biomaterials used in muscle tissue engineering include alginate [116], gelatin [93], chitosan [117] and various polysaccharide biopolymers. Other advanced biomaterials applied in engineered motor systems, like bones and cartilage, are considerable choices as well [118–120]. A key strategy in scaffold optimization involves functionalized hydrogels, which have been extensively explored for enhancing muscle tissue modeling [116, 121, 122]. For instance, Xue *et al.* [101], Wang *et al.* [123] and Saveh-Shemshaki *et al.* [124] demonstrated that conductive hydrogel materials can effectively modulate muscle tissue regeneration and differentiation. Beyond cellular scaffolds, synthetic biomaterials are also incorporated into microphysiological systems. For example, Winston *et al.* [125] discovered a novel nanoglass material capable of modulating skeletal muscle differentiation and regeneration.

## Detection and readout of signals

**Figure 7.**
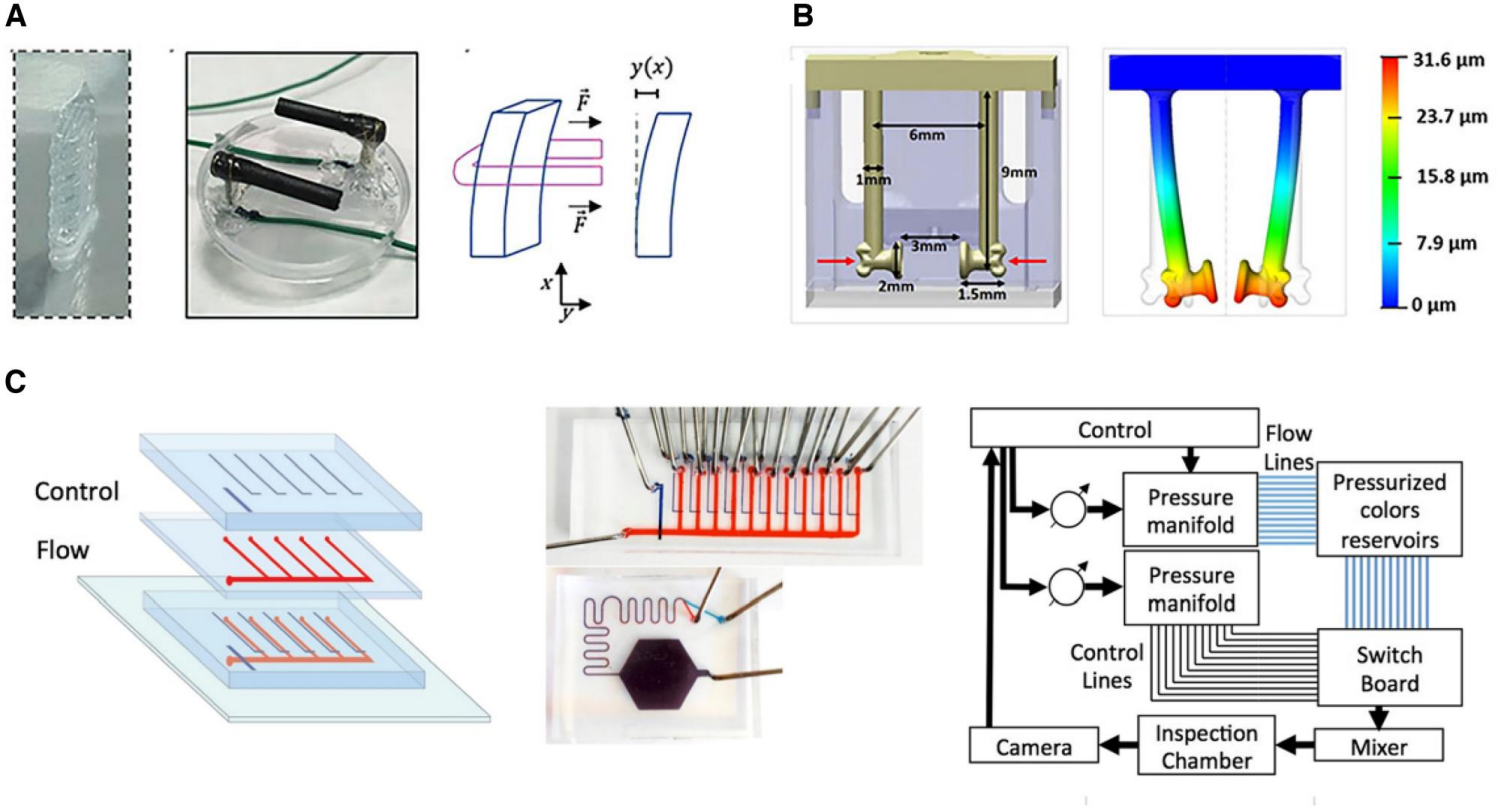
Detection of various types of signals in the microphysiological system. (**A**) Electrical stimulation triggers muscle contraction, pulling on the basal column deformation and converting tension by pre-measured modulus of elasticity [85]. (**B**) Quantitative measurements of the basal column deformation induced by the muscle pulling [126]. (**C**) Introducing a variety of sensors into the microfluidic system and participating in its modulation [127].

Following the successful construction of a microphysiological system and functional muscle tissues, diverse tools for assessing muscle tissue by detecting and reading physiological and biochemical signals have advanced researchers’ understanding of skeletal muscle function and characterization (as shown in Figure 7).

### Structural/microscopic characterization

The well-organized structure of muscle segments is fundamental to the function of skeletal muscle, making microscopic observation the first step in verifying its performance. These key structural and functional proteins, such as actin [84, 89], myosin heavy chain [87, 88] and MyoD [57, 59], are commonly labeled using immunofluorescence and immunohistochemical staining due to their high expression in skeletal muscle tissues. These markers can be used to evaluate the degree of myogenic differentiation at early and late stages.

In addition to detecting key proteins, basic structures such as the sarcoplasmic reticulum and myofibrils can be observed using transmission electron microscopy [128]. For quantitative analysis of the structural integrity of muscle bundles, a typical index, such as the nuclear fusion index [81], can be used.

### Contractile functional characterization

Calcium ions enter the myocyte from the sarcoplasmic reticulum, causing changes in actin and myosin, leading to the sliding of thick and thin myofilaments and shortening of muscle segments, resulting in muscle fiber contraction. Measuring tension is the most intuitive way to characterize contractile capacity. Due to the small size of muscle tissue in microphysiological systems, conventional tension sensors cannot be used, so stresses are usually calculated by pre-measuring the modulus of elasticity of the material, followed by measurements of the muscle bundles' attachment points or hydrogel deformations [73, 105]. In addition to tension, the rate and amplitude of muscle contraction are additional options, and cellular imaging can be utilized to record the rate and duration of muscle contraction in addition to macroscopic-level video [72, 95]. Given the varied conditions and sizes of different models, researchers defined specific force/mechanical stress as the ratio of tension to the cross-sectional area of the muscle bundle to normalize tension comparison [129]. By integrating measurements of length, velocity and contractile frequency, this approach enables a more comprehensive evaluation of contraction variations among models [129]. Incorporating these metrics allows for a thorough assessment of contraction variations across models. In addition to muscle contraction, calcium transient monitoring can also be chosen to examine the influx and outflow of calcium ions to assess muscle activity [70].

### Electrical/biochemical sensors

The excitatory signals for normal muscle physiological activity come from the NMJ, making it possible to monitor electrophysiological signals through methods such as diaphragm clamps [60] and microelectrode arrays [130]. Additionally, the microfluidic system allows for the integration of various physiological and biochemical sensors to further emulate the positive and negative feedback effects of various physiological conditions [127], e.g. integrating piezoelectric materials as high-sensitivity force sensors [131].

## Future prospects

### Physiological model construction

**Figure 8.**
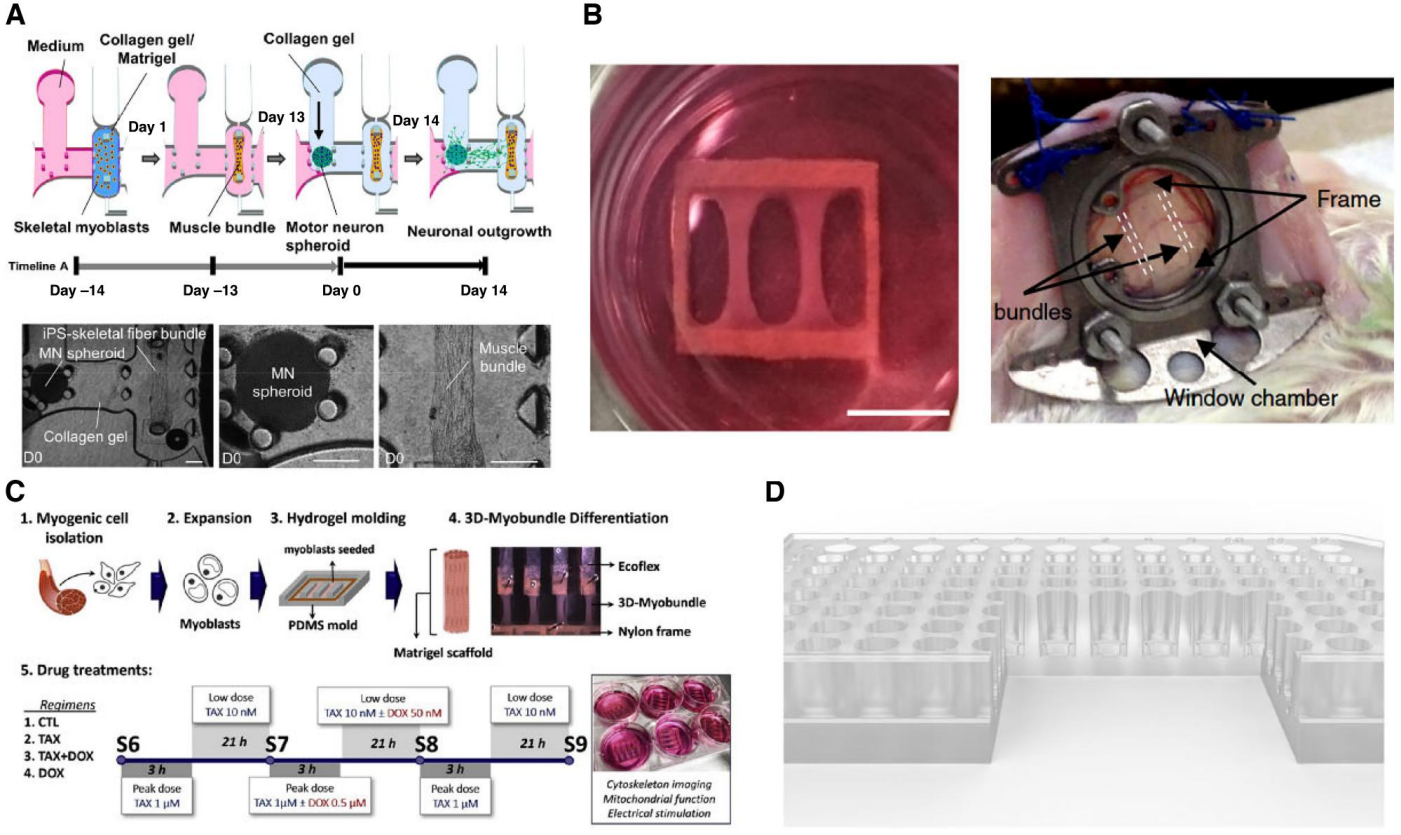
Different application scenarios of the artificial muscle microphysiology system. (**A**) Establishment of NMJ organ chips for simulating diseases such as ALS and drug screening [76]. (**B**) Cultivated artificial muscle bundles can also be reimplanted *in vivo* to validate their physiological function processes [57]. (**C**) Microchip models for validating the toxicity of chemotherapeutic drugs on muscle tissue [88]. (**D**) High-throughput muscle microchip models that can be used to carry out experiments such as drug screening in a more efficient manner [69].

Many aspects of muscle physiology remain unexplored, and current engineering strategies offer significant room for improvement. Pallotta *et al.* [71] and Giza *et al.* [77] created skeletal muscle models of varying age origins and used electrical stimulation to mimic human movement, exploring changes in the properties and composition of myofibers (as shown in Figure 8). Both Pallotta *et al.* [71] and Khodabukus *et al.* [103] found that electrical stimulation of engineered muscle led to changes in myofiber diameter, contractile force and phenotypic shift from fast-type to slow-type, highlighting its crucial role in muscle development and function and the application prospects as an alternative *in vivo* model. Gao *et al.* [52] examined cell movement patterns during myotube generation and differentiation by modifying substrate materials and patterns. Wang analysed SC heterogeneity under exercise conditions in the constructed system [90], and Chen *et al.* [104] investigated the regulation of the muscle JAK–STAT pathway by exercise. Van Der Wal *et al.* [70] compared human primary myogenic cells and iPSC-induced differentiated cells obtained from skeletal muscle and identified differences in contractility and proteomic profiles. Kondash *et al.* [132] examined the effect of insulin on glucose metabolism in muscle. Researchers such as Fernández-Garibay *et al.* [78], Armstrong *et al.* [79] and Kim *et al.* [86] refined materials and construction strategies to enhance the functionality of existing models.

### Disease modeling and pharmacotoxicological testing

Pathological and pharmacological testing of muscle remains a primary application of *in vitro* muscle modeling, highlighting the advantages of *in vitro* modeling due to the challenges and invasiveness of muscle sampling. Osaki *et al.* [75] developed an ALS disease model with patient-derived iPSC that demonstrated typical genotype, replicating the neurological and myogenic abnormalities such as neuron atrophy and force reduction, which can be observed in ALS patients, and evaluating the neuroprotective effects and muscle apoptosis reversal by potential drugs such as rapamycin and bosutinib. Afshar *et al.* [72] constructed a myasthenia gravis (MG) model with patient-derived cells, observing abnormal IgG deposition and reproducing muscle dysfunction by comparing models from healthy and patient populations. Jiwalawat *et al.* [80] and Wang *et al.* [133] replicated Pompe disease phenotypes on patterned planar cultures, while Caputo *et al.* [50] identified Duchenne muscular dystrophy (DMD) phenotypes by reprogramming and differentiating patient-derived cells, demonstrating the critical role of the TGFβ/SMAD pathway in DMD pathology. These studies underscore the feasibility of microphysiological models for mimicking muscle diseases *in vitro*. *In vitro* modeling is critical for drug screening efforts, particularly with microphysiological models enabling high-throughput and prolonged drug testing [69]. Functionalized muscle microphysiological models also enable diverse toxicological assessments. Torres *et al.* [88] successfully engineered muscle bundles and exposed them to chemotherapeutic drugs. Following treatment, these functional muscle bundles exhibited altered respiratory activity and reduction in contractile force, demonstrating a novel approach for drug testing.

### Advanced functional muscle bundles for tissue repair

Damage to the basal lamina in muscle tissue triggers the release of chelating growth factors, activating SCs to proliferate along the basal lamina and migrate to the wound site. SCs begin to fuze to form myotubes while undergoing self-renewal. In cases of severe muscle trauma, most of the native basal lamina and SCs are destroyed, diminishing SC-mediated regeneration and allowing fibroblasts to infiltrate the wound [134]. Such injuries are characterized by myoblast infiltration and sparse, disorganized collagen deposition, leading to scar tissue formation and muscle reduction [134, 135]. When muscle tissue experiences volumetric loss with severe dysfunction that exceeds its regenerative capacity, scar healing becomes the default outcome [115]. This underscores tissue engineering as a promising therapeutic option for addressing muscle tissue loss. Significant progress has been made in animal experiments [83, 84, 107]. Rao *et al.* successfully reconstructed a murine muscle bundle and implanted it *in vivo*, demonstrating the engraftment and functional capacity of myobundles [57]; Somers *et al.* [84] and Gilbert-Honick *et al.* [83] advanced this work by implanting engineered muscle tissue into a volumetric muscle loss model, ultimately observing signs of vascularization and myogenic regeneration. Carosio *et al.* [136] documented an improvement in functional recovery by up to 14% in his research. These advancements underscore the material's biocompatibility and consequently broaden our perspective on its potential applications in the human body. Furthermore, with the advancement of tendon/ligament bioengineering, the integrity of engineered muscle shall be improved, bringing us closer to application [25, 137].

### Future directions

Current models can already emulate muscle fibers with contractile function by introducing multiple cell types at the cellular level, including neural tissue, tendon/ligament tissue and vascular endothelial tissue. However, the composition of the myoblast microenvironment is highly complex, and the inclusion of other components, such as immune cells, FAPs and other mesenchymal cells, remains underexplored. As previously demonstrated, various cellular activities depend on non-myogenic cells. This limitation impedes the study of pathological alterations, such as muscle lysis, which represents a promising avenue for further research. Introducing immune cells into the microphysiological system, through either co-culture or a two-compartment culture approach, could offer a potential solution. Additionally, the involvement of FAPs shows great potential due to their multifaceted role in muscle regeneration. Establishing a co-culture system integrating myogenic cells and FAPs could serve as a valuable future direction for advancing skeletal muscle modeling.

In addition to neuromuscular and musculoligamentous connections, skeletal muscle tissue interacts with other tissues, such as bones, cartilage and skin, with their rapid advancement in tissue engineering, making these interactions a crucial target for future simulation studies. Cellular and molecular research on how myogenic cells interact with surrounding cells and biochemical factors will further drive advancements in the field. Uncovering previously unknown mechanisms shall lay the foundation for medical applications. When combined with advanced material technologies, these insights may lead to cross-disciplinary applications in areas such as wound healing and cosmetic surgery [92, 138, 139], and furthermore, serve as a significant approach in regenerative medicine.

## Summary

*In vitro* modeling technology, with continuous theoretical and technological advancements, is steadily progressing toward replicating real physiological functions at the microscopic level. Preliminary simulations have facilitated the study of skeletal muscle physiology and pathology. Pathophysiological modeling has enabled drug screening applications, offering researchers more flexibility by overcoming the limitations of animal tissues and better replicating the human environment *in vitro*. However, the current skeletal muscle microphysiology system requires further advancements to better replicate the real microenvironment and expand its application to broader fields.

## References

[1] Mukund K , Subramaniam S. Skeletal muscle: a review of molecular structure and function, in health and disease. WIREs Syst Biol Med 2020;12:e1462. 10.1002/wsbm.1462 PMC6916202 31407867

[2] Rodríguez C , Timóteo-Ferreira F, Minchiotti G, Brunelli S, Guardiola O. Cellular interactions and microenvironment dynamics in skeletal muscle regeneration and disease. Front Cell Dev Biol 2024;12:1385399. 38840849 10.3389/fcell.2024.1385399 PMC11150574

[3] Dumont NA , Wang YX, Rudnicki MA. Intrinsic and extrinsic mechanisms regulating satellite cell function. Development 2015;142:1572–81. 10.1242/dev.114223. 25922523 PMC4419274

[4] Sousa-Victor P , García-Prat L, Muñoz-Cánoves P. Control of satellite cell function in muscle regeneration and its disruption in ageing. Nat Rev Mol Cell Biol 2022;23:204–26. 34663964 10.1038/s41580-021-00421-2

[5] Guo Q , Luo Q, Song G. Control of muscle satellite cell function by specific exercise‐induced cytokines and their applications in muscle maintenance. J Cachexia Sarcopenia Muscle 2024;15:466–76. 38375571 10.1002/jcsm.13440 PMC10995279

[6] Wosczyna MN , Konishi CT, Carbajal EEP, Wang TT, Walsh RA, Gan Q, Wagner MW, Rando TA. Mesenchymal stromal cells are required for regeneration and homeostatic maintenance of skeletal muscle. Cell Rep 2019;27:2029–35.e5. 31091443 10.1016/j.celrep.2019.04.074 PMC7034941

[7] Joe AWB , Yi L, Natarajan A, Le Grand F, So L, Wang J, Rudnicki MA, Rossi FMV. Muscle injury activates resident fibro/adipogenic progenitors that facilitate myogenesis. Nat Cell Biol 2010;12:153–63. 20081841 10.1038/ncb2015 PMC4580288

[8] Kang X , Yang M, Shi Y, Xie M, Zhu M, Zheng X, Zhang C, Ge Z, Bian X, Lv J, Wang Y, Zhou B, Tang K. Interleukin-15 facilitates muscle regeneration through modulation of fibro/adipogenic progenitors. Cell Commun Signal 2018;16:42. 30029643 10.1186/s12964-018-0251-0 PMC6053744

[9] Wosczyna MN , Perez Carbajal EE, Wagner MW, Paredes S, Konishi CT, Liu L, Wang TT, Walsh RA, Gan Q, Morrissey CS, Rando TA. Targeting microRNA-mediated gene repression limits adipogenic conversion of skeletal muscle mesenchymal stromal cells. Cell Stem Cell 2021;28:1323–34.e8. 33945794 10.1016/j.stem.2021.04.008 PMC8254802

[10] Schüler SC , Liu Y, Dumontier S, Grandbois M, Le Moal E, Cornelison D, Bentzinger CF. Extracellular matrix: brick and mortar in the skeletal muscle stem cell niche. Front Cell Dev Biol 2022;10:1056523. 36523505 10.3389/fcell.2022.1056523 PMC9745096

[11] Malecova B , Gatto S, Etxaniz U, Passafaro M, Cortez A, Nicoletti C, Giordani L, Torcinaro A, De Bardi M, Bicciato S, De Santa F, Madaro L, Puri PL. Dynamics of cellular states of fibro-adipogenic progenitors during myogenesis and muscular dystrophy. Nat Commun 2018;9:3670. 30202063 10.1038/s41467-018-06068-6 PMC6131350

[12] Csapo R , Gumpenberger M, Wessner B. Skeletal muscle extracellular matrix—what do we know about its composition, regulation, and physiological roles? A narrative review. Front Physiol 2020;11:253. 32265741 10.3389/fphys.2020.00253 PMC7096581

[13] Baghdadi MB , Castel D, Machado L, Fukada S-I, Birk DE, Relaix F, Tajbakhsh S, Mourikis P. Reciprocal signalling by Notch-Collagen V-CALCR retains muscle stem cells in their niche. Nature 2018;557:714–8. 29795344 10.1038/s41586-018-0144-9 PMC5985950

[14] Loreti M , Sacco A. The jam session between muscle stem cells and the extracellular matrix in the tissue microenvironment. npj Regen Med 2022;7:16. 35177651 10.1038/s41536-022-00204-z PMC8854427

[15] de Almeida PG , Pinheiro GG, Nunes AM, Gonçalves AB, Thorsteinsdóttir S. Fibronectin assembly during early embryo development: a versatile communication system between cells and tissues. Dev Dyn Off Publ Am Assoc Anat 2016;245:520–35. 10.1002/dvdy.24391 26845241

[16] Bentzinger CF , Wang YX, von Maltzahn J, Soleimani VD, Yin H, Rudnicki MA. Fibronectin regulates Wnt7a signaling and satellite cell expansion. Cell Stem Cell 2013;12:75–87. 23290138 10.1016/j.stem.2012.09.015 PMC3539137

[17] Roman W , Martins JP, Gomes ER. Local arrangement of fibronectin by myofibroblasts governs peripheral nuclear positioning in muscle cells. Dev Cell 2018;46:102–11.e6. 29937388 10.1016/j.devcel.2018.05.031 PMC6035285

[18] Rozo M , Li L, Fan C-M. Targeting β1-integrin signaling enhances regeneration in aged and dystrophic muscle in mice. Nat Med 2016;22:889–96. 27376575 10.1038/nm.4116 PMC4974124

[19] Nunes AM , Wuebbles RD, Sarathy A, Fontelonga TM, Deries M, Burkin DJ, Thorsteinsdóttir S. Impaired fetal muscle development and JAK-STAT activation mark disease onset and progression in a mouse model for merosin-deficient congenital muscular dystrophy. Hum Mol Genet 2017;26:2018–33. 28334989 10.1093/hmg/ddx083 PMC6075618

[20] Rayagiri SS , Ranaldi D, Raven A, Mohamad Azhar NIF, Lefebvre O, Zammit PS, Borycki A-G. Basal lamina remodeling at the skeletal muscle stem cell niche mediates stem cell self-renewal. Nat Commun 2018;9:1075. 29540680 10.1038/s41467-018-03425-3 PMC5852002

[21] De Almeida LGN , Thode H, Eslambolchi Y, Chopra S, Young D, Gill S, Devel L, Dufour A. Matrix metalloproteinases: from molecular mechanisms to physiology, pathophysiology, and pharmacology. Pharmacol Rev 2022;74:714–70. 10.1124/pharmrev.121.000349 35738680

[22] Zhang J , Si R, Gao Y, Shan H, Su Q, Feng Z, Huang P, Kong D, Wang W. dECM restores macrophage immune homeostasis and alleviates iron overload to promote DTPI healing. Regen Biomater 2024;11:rbad118. 38404617 10.1093/rb/rbad118 PMC10884736

[23] Xu P , Kankala RK, Wang S, Chen A. Decellularized extracellular matrix-based composite scaffolds for tissue engineering and regenerative medicine. Regen Biomater 2024;11:rbad107. 38173774 10.1093/rb/rbad107 PMC10761212

[24] Chen W , Chen M, Chen S, Wang S, Huang Z, Zhang L, Wu J, Peng W, Li H, Wen F. Decellularization of fish tissues for tissue engineering and regenerative medicine applications. Regen Biomater 2025;12:rbae138. 39776859 10.1093/rb/rbae138 PMC11703550

[25] Ning L-J , Cui J, He S-K, Hu R-N, Yao X, Zhang Y, Ding W, Zhang Y-J, Luo J-C, Qin T-W. Constructing a highly bioactive tendon-regenerative scaffold by surface modification of tissue-specific stem cell-derived extracellular matrix. Regen Biomater 2022;9:rbac020. 35480863 10.1093/rb/rbac020 PMC9036902

[26] Uderhardt S , Martins AJ, Tsang JS, Lämmermann T, Germain RN. Resident macrophages cloak tissue microlesions to prevent neutrophil-driven inflammatory damage. Cell 2019;177:541–55.e17. 30955887 10.1016/j.cell.2019.02.028 PMC6474841

[27] Wang X , Sathe AA, Smith GR, Ruf-Zamojski F, Nair V, Lavine KJ, Xing C, Sealfon SC, Zhou L. Heterogeneous origins and functions of mouse skeletal muscle-resident macrophages. Proc Natl Acad Sci U S A 2020;117:20729–40. 32796104 10.1073/pnas.1915950117 PMC7456122

[28] Henrot P , Blervaque L, Dupin I, Zysman M, Esteves P, Gouzi F, Hayot M, Pomiès P, Berger P. Cellular interplay in skeletal muscle regeneration and wasting: insights from animal models. J Cachexia Sarcopenia Muscle 2023;14:745–57. 36811134 10.1002/jcsm.13103 PMC10067506

[29] Montero D , Cathomen A, Jacobs RA, Flück D, De Leur J, Keiser S, Bonne T, Kirk N, Lundby A, Lundby C. Haematological rather than skeletal muscle adaptations contribute to the increase in peak oxygen uptake induced by moderate endurance training. J Physiol 2015;593:4677–88. 26282186 10.1113/JP270250 PMC4606528

[30] Attwell D , Mishra A, Hall CN, O'Farrell FM, Dalkara T. What is a pericyte? J Cereb Blood Flow Metab Off J Int Soc Cereb Blood Flow Metab 2016;36:451–5. 10.1177/0271678X15610340 PMC4759679 26661200

[31] Wu Y-F , Lapp S, Dvoretskiy S, Garcia G, Kim M, Tannehill A, Daniels L, Boppart MD. Optimization of a pericyte therapy to improve muscle recovery after limb immobilization. J Appl Physiol Bethesda Md 1985 2022;132:1020–30. 10.1152/japplphysiol.00700.2021 PMC8993526 35175105

[32] Ceafalan LC , Fertig TE, Popescu AC, Popescu BO, Hinescu ME, Gherghiceanu M. Skeletal muscle regeneration involves macrophage-myoblast bonding. Cell Adhes Migr 2018;12:228–35. 10.1080/19336918.2017.1346774 PMC6149487 28759306

[33] Varga T , Mounier R, Horvath A, Cuvellier S, Dumont F, Poliska S, Ardjoune H, Juban G, Nagy L, Chazaud B. Highly dynamic transcriptional signature of distinct macrophage subsets during sterile inflammation, resolution, and tissue repair. J Immunol 2016;196:4771–82. 27183604 10.4049/jimmunol.1502490

[34] Juhas M , Abutaleb N, Wang JT, Ye J, Shaikh Z, Sriworarat C, Qian Y, Bursac N. Incorporation of macrophages into engineered skeletal muscle enables enhanced muscle regeneration. Nat Biomed Eng 2018;2:942–54. 30581652 10.1038/s41551-018-0290-2 PMC6296488

[35] Andre AB , Rees KP, O'Connor S, Severson GW, Newbern JM, Wilson-Rawls J, Plaisier CL, Rawls A. Single cell analysis reveals satellite cell heterogeneity for proinflammatory chemokine expression. Front Cell Dev Biol 2023;11:1084068. 37051469 10.3389/fcell.2023.1084068 PMC10083252

[36] Fiore D , Judson RN, Low M, Lee S, Zhang E, Hopkins C, Xu P, Lenzi A, Rossi FMV, Lemos DR. Pharmacological blockage of fibro/adipogenic progenitor expansion and suppression of regenerative fibrogenesis is associated with impaired skeletal muscle regeneration. Stem Cell Res 2016;17:161–9. 27376715 10.1016/j.scr.2016.06.007

[37] Juban G , Saclier M, Yacoub-Youssef H, Kernou A, Arnold L, Boisson C, Ben Larbi S, Magnan M, Cuvellier S, Théret M, Petrof BJ, Desguerre I, Gondin J, Mounier R, Chazaud B. AMPK activation regulates LTBP4-dependent TGF-β1 secretion by pro-inflammatory macrophages and controls fibrosis in Duchenne muscular dystrophy. Cell Rep 2018;25:2163–76.e6. 30463013 10.1016/j.celrep.2018.10.077

[38] Mázala DAG , Hindupur R, Moon YJ, Shaikh F, Gamu IH, Alladi D, Panci G, Weiss-Gayet M, Chazaud B, Partridge TA, Novak JS, Jaiswal JK. Altered muscle niche contributes to myogenic deficit in the D2-mdx model of severe DMD. *Cell Death Discov* **2023**;9:224. 10.1038/s41420-023-01503-0 PMC10319851 37402716

[39] Kotsaris G , Qazi TH, Bucher CH, Zahid H, Pöhle-Kronawitter S, Ugorets V, Jarassier W, Börno S, Timmermann B, Giesecke-Thiel C, Economides AN, Le Grand F, Vallecillo-García P, Knaus P, Geissler S, Stricker S. Odd skipped-related 1 controls the pro-regenerative response of fibro-adipogenic progenitors. npj Regen Med 2023;8:19. 37019910 10.1038/s41536-023-00291-6 PMC10076435

[40] Christov C , Chrétien F, Abou-Khalil R, Bassez G, Vallet G, Authier F-J, Bassaglia Y, Shinin V, Tajbakhsh S, Chazaud B, Gherardi RK. Muscle satellite cells and endothelial cells: close neighbors and privileged partners. Mol Biol Cell 2007;18:1397–409. 17287398 10.1091/mbc.E06-08-0693 PMC1838982

[41] Latroche C , Weiss-Gayet M, Muller L, Gitiaux C, Leblanc P, Liot S, Ben-Larbi S, Abou-Khalil R, Verger N, Bardot P, Magnan M, Chrétien F, Mounier R, Germain S, Chazaud B. Coupling between myogenesis and angiogenesis during skeletal muscle regeneration is stimulated by restorative macrophages. Stem Cell Rep 2017;9:2018–33. 10.1016/j.stemcr.2017.10.027 PMC5785732 29198825

[42] Leng Y , Li X, Zheng F, Liu H, Wang C, Wang X, Liao Y, Liu J, Meng K, Yu J, Zhang J, Wang B, Tan Y, Liu M, Jia X, Li D, Li Y, Gu Z, Fan Y. Advances in in vitro models of neuromuscular junction: focusing on organ‐on‐a‐chip, organoids, and biohybrid robotics. Adv Mater 2023;35:2211059. 10.1002/adma.202211059 36934404

[43] Hinkle ER , Blue RE, Tsai Y-H, Combs M, Davi J, Coffey AR, Boriek AM, Taylor JM, Parker JS, Giudice J. Stretching muscle cells induces transcriptional and splicing transitions and changes in SR proteins. Commun Biol 2022;5:987. 36123433 10.1038/s42003-022-03915-7 PMC9485123

[44] Kim J , Park I, Shin HR, Rhee J, Seo J, Jo Y, Yoo K, Hann S, Kang J, Park J, Kim YL, Moon J, Choi MH, Kong Y. The hypothalamic–pituitary–gonadal axis controls muscle stem cell senescence through autophagosome clearance. J Cachexia Sarcopenia Muscle 2021;12:177–91. 33244887 10.1002/jcsm.12653 PMC7890269

[45] LeBlanc L , Ramirez N, Kim J. Context-dependent roles of YAP/TAZ in stem cell fates and cancer. Cell Mol Life Sci 2021;78:4201–19. 33582842 10.1007/s00018-021-03781-2 PMC8164607

[46] Chal J , Oginuma M, Al Tanoury Z, Gobert B, Sumara O, Hick A, Bousson F, Zidouni Y, Mursch C, Moncuquet P, Tassy O, Vincent S, Miyanari A, Bera A, Garnier J-M, Guevara G, Hestin M, Kennedy L, Hayashi S, Drayton B, Cherrier T, Gayraud-Morel B, Gussoni E, Relaix F, Tajbakhsh S, Pourquié O. Differentiation of pluripotent stem cells to muscle fiber to model Duchenne muscular dystrophy. Nat Biotechnol 2015;33:962–9. 26237517 10.1038/nbt.3297

[47] Andersen J , Revah O, Miura Y, Thom N, Amin ND, Kelley KW, Singh M, Chen X, Thete MV, Walczak EM, Vogel H, Fan HC, Paşca SP. Generation of functional human 3D cortico-motor assembloids. Cell 2020;183:1913–29.e26. 33333020 10.1016/j.cell.2020.11.017 PMC8711252

[48] Shin M-K , Bang JS, Lee JE, Tran H-D, Park G, Lee DR, Jo J. Generation of skeletal muscle organoids from human pluripotent stem cells to model myogenesis and muscle regeneration. Int J Mol Sci 2022;23:5108. 35563499 10.3390/ijms23095108 PMC9103168

[49] Abujarour R , Bennett M, Valamehr B, Lee TT, Robinson M, Robbins D, Le T, Lai K, Flynn P. Myogenic differentiation of muscular dystrophy-specific induced pluripotent stem cells for use in drug discovery. Stem Cells Transl Med 2014;3:149–60. 24396035 10.5966/sctm.2013-0095 PMC3925053

[50] Caputo L , Granados A, Lenzi J, Rosa A, Ait-Si-Ali S, Puri PL, Albini S. Acute conversion of patient-derived Duchenne muscular dystrophy iPSC into myotubes reveals constitutive and inducible over-activation of TGFβ-dependent pro-fibrotic signaling. Skelet Muscle 2020;10:13. 32359374 10.1186/s13395-020-00224-7 PMC7195779

[51] Iacovino M , Bosnakovski D, Fey H, Rux D, Bajwa G, Mahen E, Mitanoska A, Xu Z, Kyba M. Inducible cassette exchange: a rapid and efficient system enabling conditional gene expression in embryonic stem and primary cells. Stem Cells 2011;29:1580–8. 22039605 10.1002/stem.715 PMC3622722

[52] Gao J , Sun X, Ma Y, Qin W, Li J, Jin Z, Qiu J, Zhang H. Myotube formation on micropatterns guiding by centripetal cellular motility and crowding. Mater Today Bio 2024;28:101195. 10.1016/j.mtbio.2024.101195 PMC11357802 39205872

[53] Coren L , Zaffryar-Eilot S, Odeh A, Kaganovsky A, Hasson P. Fibroblast diversification is an embryonic process dependent on muscle contraction. Cell Rep 2024;43:115034. 39636726 10.1016/j.celrep.2024.115034

[54] Gilbert PM , Havenstrite KL, Magnusson KEG, Sacco A, Leonardi NA, Kraft P, Nguyen NK, Thrun S, Lutolf MP, Blau HM. Substrate elasticity regulates skeletal muscle stem cell self-renewal in culture. Science 2010;329:1078–81. 20647425 10.1126/science.1191035 PMC2929271

[55] Maffioletti SM , Sarcar S, Henderson ABH, Mannhardt I, Pinton L, Moyle LA, Steele-Stallard H, Cappellari O, Wells KE, Ferrari G, Mitchell JS, Tyzack GE, Kotiadis VN, Khedr M, Ragazzi M, Wang W, Duchen MR, Patani R, Zammit PS, Wells DJ, Eschenhagen T, Tedesco FS. Three-dimensional human iPSC-derived artificial skeletal muscles model muscular dystrophies and enable multilineage tissue engineering. Cell Rep 2018;23:899–908. 29669293 10.1016/j.celrep.2018.03.091 PMC5917451

[56] Volpi M , Paradiso A, Walejewska E, Gargioli C, Costantini M, Swieszkowski W. Automated microfluidics-assisted hydrogel-based wet-spinning for the biofabrication of biomimetic engineered myotendinous junction. Adv Healthc Mater 2024;13:e2402075. 39313990 10.1002/adhm.202402075 PMC11670271

[57] Rao L , Qian Y, Khodabukus A, Ribar T, Bursac N. Engineering human pluripotent stem cells into a functional skeletal muscle tissue. Nat Commun 2018;9:126. 29317646 10.1038/s41467-017-02636-4 PMC5760720

[58] Zhao M , Shoji E, Sakurai H. In vitro evaluation of exon skipping in disease-specific iPSC-derived myocytes. In: Yokota T, Maruyama R (eds.). Exon Skipping and Inclusion Therapies: Methods and Protocols. New York, NY: Springer, 2018, 173–89. 10.1007/978-1-4939-8651-4_11 30171542

[59] Shahriyari M , Islam MR, Sakib SM, Rinn M, Rika A, Krüger D, Kaurani L, Gisa V, Winterhoff M, Anandakumar H, Shomroni O, Schmidt M, Salinas G, Unger A, Linke WA, Zschüntzsch J, Schmidt J, Bassel‐Duby R, Olson EN, Fischer A, Zimmermann W, Tiburcy M. Engineered skeletal muscle recapitulates human muscle development, regeneration and dystrophy. J Cachexia Sarcopenia Muscle 2022;13:3106–21. 36254806 10.1002/jcsm.13094 PMC9745484

[60] Mavrommatis L , Jeong H-W, Kindler U, Gomez-Giro G, Kienitz M-C, Stehling M, Psathaki OE, Zeuschner D, Bixel MG, Han D, Morosan-Puopolo G, Gerovska D, Yang JH, Kim JB, Arauzo-Bravo MJ, Schwamborn JC, Hahn SA, Adams RH, Schöler HR, Vorgerd M, Brand-Saberi B, Zaehres H. Human skeletal muscle organoids model fetal myogenesis and sustain uncommitted PAX7 myogenic progenitors. eLife 2023;12:RP87081. 37963071 10.7554/eLife.87081 PMC10645425

[61] Faustino Martins J-M , Fischer C, Urzi A, Vidal R, Kunz S, Ruffault P-L, Kabuss L, Hube I, Gazzerro E, Birchmeier C, Spuler S, Sauer S, Gouti M. Self-organizing 3D human trunk neuromuscular organoids. Cell Stem Cell 2020;26:172–86.e6. 31956040 10.1016/j.stem.2019.12.007

[62] Minne M , Terrie L, Wüst R, Hasevoets S, Vanden Kerchove K, Nimako K, Lambrichts I, Thorrez L, Declercq H. Generating human skeletal myoblast spheroids for vascular myogenic tissue engineering. Biofabrication 2024;16:025035. 10.1088/1758-5090/ad2fd5 38437715

[63] Bosutti A , Giniatullin A, Odnoshivkina Y, Giudice L, Malm T, Sciancalepore M, Giniatullin R, D’Andrea P, Lorenzon P, Bernareggi A. “Time window” effect of Yoda1‐evoked Piezo1 channel activity during mouse skeletal muscle differentiation. Acta Physiol 2021;233:e13702. 10.1111/apha.13702 PMC9286833 34097801

[64] Hirano K , Tsuchiya M, Shiomi A, Takabayashi S, Suzuki M, Ishikawa Y, Kawano Y, Takabayashi Y, Nishikawa K, Nagao K, Umemoto E, Kitajima Y, Ono Y, Nonomura K, Shintaku H, Mori Y, Umeda M, Hara Y. The mechanosensitive ion channel PIEZO1 promotes satellite cell function in muscle regeneration. Life Sci Alliance 2023;6:e202201783. 36446523 10.26508/lsa.202201783 PMC9711862

[65] Jalal S , Dastidar S, Tedesco FS. Advanced models of human skeletal muscle differentiation, development and disease: three-dimensional cultures, organoids and beyond. Curr Opin Cell Biol 2021;73:92–104. 34384976 10.1016/j.ceb.2021.06.004 PMC8692266

[66] Hinds S , Bian W, Dennis RG, Bursac N. The role of extracellular matrix composition in structure and function of bioengineered skeletal muscle. Biomaterials 2011;32:3575–83. 21324402 10.1016/j.biomaterials.2011.01.062 PMC3057410

[67] Madden L , Juhas M, Kraus WE, Truskey GA, Bursac N. Bioengineered human myobundles mimic clinical responses of skeletal muscle to drugs. eLife 2015;4:e04885. 25575180 10.7554/eLife.04885 PMC4337710

[68] Khodabukus A , Prabhu NK, Roberts T, Buldo M, Detwiler A, Fralish ZD, Kondash ME, Truskey GA, Koves TR, Bursac N. Bioengineered model of human LGMD2B skeletal muscle reveals roles of intracellular calcium overload in contractile and metabolic dysfunction in dysferlinopathy. Adv Sci 2024;11:2400188. 10.1002/advs.202400188 PMC11336985 38887849

[69] Afshar ME , Abraha HY, Bakooshli MA, Davoudi S, Thavandiran N, Tung K, Ahn H, Ginsberg HJ, Zandstra PW, Gilbert PM. A 96-well culture platform enables longitudinal analyses of engineered human skeletal muscle microtissue strength. Sci Rep 2020;10:6918. 32332853 10.1038/s41598-020-62837-8 PMC7181829

[70] Van Der Wal E , Iuliano A, In 't Groen SLM, Bholasing AP, Priesmann D, Sharma P, Den Hamer B, Saggiomo V, Krüger M, Pijnappel WWMP, De Greef JC. Highly contractile 3D tissue engineered skeletal muscles from human iPSCs reveal similarities with primary myoblast-derived tissues. Stem Cell Rep 2023;18:1954–71. 10.1016/j.stemcr.2023.08.014 PMC10656354 37774701

[71] Pallotta I , Stec MJ, Schriver B, Golann DR, Considine K, Su Q, Barahona V, Napolitano JE, Stanley S, Garcia M, Feric NT, Durney KM, Aschar‐Sobbi R, Bays N, Shavlakadze T, Graziano MP. Electrical stimulation of biofidelic engineered muscle enhances myotube size, force, fatigue resistance, and induces a fast‐to‐slow‐phenotype shift. Physiol Rep 2024;12:e70051. 39384537 10.14814/phy2.70051 PMC11464147

[72] Afshar Bakooshli M , Lippmann ES, Mulcahy B, Iyer N, Nguyen CT, Tung K, Stewart BA, Van Den Dorpel H, Fuehrmann T, Shoichet M, Bigot A, Pegoraro E, Ahn H, Ginsberg H, Zhen M, Ashton RS, Gilbert PM. A 3D culture model of innervated human skeletal muscle enables studies of the adult neuromuscular junction. eLife 2019;8:e44530. 31084710 10.7554/eLife.44530 PMC6516829

[73] Agrawal G , Aung A, Varghese S. Skeletal muscle-on-a-chip: an in vitro model to evaluate tissue formation and injury. Lab Chip 2017;17:3447–61. 28871305 10.1039/c7lc00512a PMC6296378

[74] Kim W , Kim J, Park H-S, Jeon J. Development of microfluidic stretch system for studying recovery of damaged skeletal muscle cells. Micromachines 2018;9:671. 30567359 10.3390/mi9120671 PMC6315523

[75] Osaki T , Uzel SGM, Kamm RD. Microphysiological 3D model of amyotrophic lateral sclerosis (ALS) from human iPS-derived muscle cells and optogenetic motor neurons. Sci Adv 2018;4:eaat5847. 30324134 10.1126/sciadv.aat5847 PMC6179377

[76] Osaki T , Uzel SGM, Kamm RD. On-chip 3D neuromuscular model for drug screening and precision medicine in neuromuscular disease. Nat Protoc 2020;15:421–49. 10.1038/s41596-019-0248-1 31932771

[77] Giza S , Mojica‐Santiago JA, Parafati M, Malany LK, Platt D, Schmidt CE, Coen PM, Malany S. Microphysiological system for studying contractile differences in young, active, and old, sedentary adult derived skeletal muscle cells. Aging Cell 2022;21:e13650. 35653714 10.1111/acel.13650 PMC9282836

[78] Fernández-Garibay X , Gómez-Florit M, Domingues RMA, Gomes ME, Fernández-Costa JM, Ramón-Azcón J. Xeno-free bioengineered human skeletal muscle tissue using human platelet lysate-based hydrogels. Biofabrication 2022;14:045015. 10.1088/1758-5090/ac8dc8 36041422

[79] Armstrong JPK , Puetzer JL, Serio A, Guex AG, Kapnisi M, Breant A, Zong Y, Assal V, Skaalure SC, King O, Murty T, Meinert C, Franklin AC, Bassindale PG, Nichols MK, Terracciano CM, Hutmacher DW, Drinkwater BW, Klein TJ, Perriman AW, Stevens MM. Engineering anisotropic muscle tissue using acoustic cell patterning. Adv Mater 2018;30:e1802649. 30277617 10.1002/adma.201802649 PMC6386124

[80] Jiwlawat N , Lynch EM, Napiwocki BN, Stempien A, Ashton RS, Kamp TJ, Crone WC, Suzuki M. Micropatterned substrates with physiological stiffness promote cell maturation and Pompe disease phenotype in human induced pluripotent stem cell‐derived skeletal myocytes. Biotechnol Bioeng 2019;116:2377–92. 31131875 10.1002/bit.27075 PMC6699746

[81] Fernández-Garibay X , Ortega MA, Cerro-Herreros E, Comelles J, Martínez E, Artero R, Fernández-Costa JM, Ramón-Azcón J. Bioengineered in vitro 3D model of myotonic dystrophy type 1 human skeletal muscle. Biofabrication 2021;13:035035. 10.1088/1758-5090/abf6ae 33836519

[82] Al Tanoury Z , Zimmerman JF, Rao J, Sieiro D, McNamara HM, Cherrier T, Rodríguez-delaRosa A, Hick-Colin A, Bousson F, Fugier-Schmucker C, Marchiano F, Habermann B, Chal J, Nesmith AP, Gapon S, Wagner E, Gupta VA, Bassel-Duby R, Olson EN, Cohen AE, Parker KK, Pourquié O. Prednisolone rescues Duchenne muscular dystrophy phenotypes in human pluripotent stem cell-derived skeletal muscle in vitro. Proc Natl Acad Sci U S A 2021;118:e2022960118. 34260377 10.1073/pnas.2022960118 PMC8285911

[83] Gilbert-Honick J , Ginn B, Zhang Y, Salehi S, Wagner KR, Mao H-Q, Grayson WL. Adipose-derived stem/stromal cells on electrospun fibrin microfiber bundles enable moderate muscle reconstruction in a volumetric muscle loss model. Cell Transplant 2018;27:1644–56. 30298751 10.1177/0963689718805370 PMC6299198

[84] Somers SM , Gilbert-Honick J, Choi IY, Lo EKW, Lim HT, Dias S, Wagner KR, Mao H-Q, Cahan P, Lee G, Grayson WL. Engineering skeletal muscle grafts with PAX7::GFP-sorted human pluripotent stem dell-derived myogenic progenitors on fibrin microfiber bundles for tissue regeneration. Bioengineering 2022;9:693. 36421094 10.3390/bioengineering9110693 PMC9687588

[85] Mestre R , Patiño T, Barceló X, Anand S, Pérez‐Jiménez A, Sánchez S. Force modulation and adaptability of 3D‐bioprinted biological actuators based on skeletal muscle tissue. Adv Mater Technol 2019;4:1800631.

[86] Kim JH , Kim I, Seol Y-J, Ko IK, Yoo JJ, Atala A, Lee SJ. Neural cell integration into 3D bioprinted skeletal muscle constructs accelerates restoration of muscle function. Nat Commun 2020;11:1025. 32094341 10.1038/s41467-020-14930-9 PMC7039897

[87] Kim J , Lee H, Lee G, Ryu D, Kim G. Fabrication of fully aligned self-assembled cell-laden collagen filaments for tissue engineering via a hybrid bioprinting process. Bioact Mater 2024;36:14–29. 38425743 10.1016/j.bioactmat.2024.02.020 PMC10900255

[88] Torres MJ , Zhang X, Slentz DH, Koves TR, Patel H, Truskey GA, Muoio DM. Chemotherapeutic drug screening in 3D-Bioengineered human myobundles provides insight into taxane-induced myotoxicities. iScience 2022;25:105189. 36274957 10.1016/j.isci.2022.105189 PMC9579017

[89] Oliver CE , Patel H, Hong J, Carter J, Kraus WE, Huffman KM, Truskey GA. Tissue engineered skeletal muscle model of rheumatoid arthritis using human primary skeletal muscle cells. J Tissue Eng Regen Med 2022;16:128–39. 34781416 10.1002/term.3266 PMC9487182

[90] Wang J , Broer T, Chavez T, Zhou CJ, Tran S, Xiang Y, Khodabukus A, Diao Y, Bursac N. Myoblast deactivation within engineered human skeletal muscle creates a transcriptionally heterogeneous population of quiescent satellite-like cells. Biomaterials 2022;284:121508. 35421801 10.1016/j.biomaterials.2022.121508 PMC9289780

[91] Dong Z , Han W, Jiang P, Hao L, Fu X. Regulation of mitochondrial network architecture and function in mesenchymal stem cells by micropatterned surfaces. Regen Biomater 2024;11:rbae052. 38854681 10.1093/rb/rbae052 PMC11162196

[92] Li J , Liu X, Tao W, Li Y, Du Y, Zhang S. Micropatterned composite membrane guides oriented cell growth and vascularization for accelerating wound healing. Regen Biomater 2023;10:rbac108. 36683746 10.1093/rb/rbac108 PMC9847515

[93] Bettadapur A , Suh GC, Geisse NA, Wang ER, Hua C, Huber HA, Viscio AA, Kim JY, Strickland JB, McCain ML. Prolonged culture of aligned skeletal myotubes on micromolded gelatin hydrogels. Sci Rep 2016;6:28855. 27350122 10.1038/srep28855 PMC4924097

[94] Kroehne V , Heschel I, Schügner F, Lasrich D, Bartsch JW, Jockusch H. Use of a novel collagen matrix with oriented pore structure for muscle cell differentiation in cell culture and in grafts. J Cell Mol Med 2008;12:1640–8. 18194451 10.1111/j.1582-4934.2008.00238.x PMC2680279

[95] Santhanam N , Kumanchik L, Guo X, Sommerhage F, Cai Y, Jackson M, Martin C, Saad G, McAleer CW, Wang Y, Lavado A, Long CJ, Hickman JJ. Stem cell derived phenotypic human neuromuscular junction model for dose response evaluation of therapeutics. Biomaterials 2018;166:64–78. 29547745 10.1016/j.biomaterials.2018.02.047 PMC5866791

[96] Lin W , Lan W, Wu Y, Zhao D, Wang Y, He X, Li J, Li Z, Luo F, Tan H, Fu Q. Aligned 3D porous polyurethane scaffolds for biological anisotropic tissue regeneration. Regen Biomater 2020;7:19–27. 32440358 10.1093/rb/rbz031 PMC7233617

[97] Yin N , Song T, Wu J, Chen B, Ma H, Zhao Z, Wang Y, Li H, Wu D. Unilateral microform cleft lip repair: application of muscle tension line group theory. J Craniofac Surg 2015;26:343–6. 25723667 10.1097/SCS.0000000000001460

[98] Yang Y , Sun J, Liu X, Guo Z, He Y, Wei D, Zhong M, Guo L, Fan H, Zhang X. Wet-spinning fabrication of shear-patterned alginate hydrogel microfibers and the guidance of cell alignment. Regen Biomater 2017;4:299–307. 29026644 10.1093/rb/rbx017 PMC5633694

[99] Kim S , Ayan B, Shayan M, Rando TA, Huang NF. Skeletal muscle-on-a-chip in microgravity as a platform for regeneration modeling and drug screening. Stem Cell Rep 2024;19:1061–73. 10.1016/j.stemcr.2024.06.010 PMC11368695 39059375

[100] Zhang M , Wang Z, Zhang A, Liu L, Mithieux SM, Bilek MMM, Weiss AS. Development of tropoelastin-functionalized anisotropic PCL scaffolds for musculoskeletal tissue engineering. Regen Biomater 2023;10:rbac087. 36683733 10.1093/rb/rbac087 PMC9845519

[101] Xue J , Qin C, Wu C. 3D printing of cell-delivery scaffolds for tissue regeneration. Regen Biomater 2023;10:rbad032. 37081861 10.1093/rb/rbad032 PMC10112960

[102] Kiratitanaporn W , Guan J, Tang M, Xiang Y, Lu T, Balayan A, Lao A, Berry DB, Chen S. 3D printing of a biomimetic myotendinous junction assisted by artificial intelligence. Biomater Sci 2024;12:6047–62. 39446075 10.1039/d4bm00892h

[103] Khodabukus A , Madden L, Prabhu NK, Koves TR, Jackman CP, Muoio DM, Bursac N. Electrical stimulation increases hypertrophy and metabolic flux in tissue-engineered human skeletal muscle. Biomaterials 2019;198:259–69. 30180985 10.1016/j.biomaterials.2018.08.058 PMC6395553

[104] Chen Z , Li B, Zhan R-Z, Rao L, Bursac N. Exercise mimetics and JAK inhibition attenuate IFN-γ-induced wasting in engineered human skeletal muscle. Sci Adv 2021;7:eabd9502. 33523949 10.1126/sciadv.abd9502 PMC10964957

[105] Khodabukus A , Kaza A, Wang J, Prabhu N, Goldstein R, Vaidya VS, Bursac N. Tissue-engineered human myobundle system as a platform for evaluation of skeletal muscle injury biomarkers. Toxicol Sci Off J Soc Toxicol 2020;176:124–36. 10.1093/toxsci/kfaa049 PMC7643536 32294208

[106] Aung A , Bhullar IS, Theprungsirikul J, Davey SK, Lim HL, Chiu Y-J, Ma X, Dewan S, Lo Y-H, McCulloch A, Varghese S. 3D cardiac μtissues within a microfluidic device with real-time contractile stress readout. Lab Chip 2016;16:153–62. 26588203 10.1039/c5lc00820d PMC4681661

[107] Juhas M , Engelmayr GC, Fontanella AN, Palmer GM, Bursac N. Biomimetic engineered muscle with capacity for vascular integration and functional maturation in vivo. Proc Natl Acad Sci U S A 2014;111:5508–13. 24706792 10.1073/pnas.1402723111 PMC3992675

[108] Ren Z , Ahn EH, Do M, Mair DB, Monemianesfahani A, Lee PHU, Kim D-H. Simulated microgravity attenuates myogenesis and contractile function of 3D engineered skeletal muscle tissues. NPJ Microgravity 2024;10:18. 38365862 10.1038/s41526-024-00353-z PMC10873406

[109] Liberman M , Chavez M, Nash TR, Vila OF, Vunjak-Novakovic G. Engineering and characterization of an optogenetic model of the human neuromuscular junction. J Vis Exp 2022:10.3791/63759. 10.3791/63759 PMC10061228 35499350

[110] Vila OF , Chavez M, Ma SP, Yeager K, Zholudeva LV, Colón-Mercado JM, Qu Y, Nash TR, Lai C, Feliciano CM, Carter M, Kamm RD, Judge LM, Conklin BR, Ward ME, McDevitt TC, Vunjak-Novakovic G. Bioengineered optogenetic model of human neuromuscular junction. Biomaterials 2021;276:121033. 34403849 10.1016/j.biomaterials.2021.121033 PMC8439334

[111] Alsaykhan H , Paxton JZ. Investigating materials and orientation parameters for the creation of a 3D musculoskeletal interface co-culture model. Regen Biomater 2020;7:413–25. 32793386 10.1093/rb/rbaa018 PMC7415002

[112] Liao J , Xu B, Zhang R, Fan Y, Xie H, Li X. Applications of decellularized materials in tissue engineering: advantages, drawbacks and current improvements, and future perspectives. J Mater Chem B 2020;8:10023–49. 33053004 10.1039/d0tb01534b

[113] Zhou H , Li W, Pan L, Zhu T, Zhou T, Xiao E, Wei Q. Human extracellular matrix (ECM)-like collagen and its bioactivity. Regen Biomater 2024;11:rbae008. 38545260 10.1093/rb/rbae008 PMC10965421

[114] Camman M , Joanne P, Brun J, Marcellan A, Dumont J, Agbulut O, Hélary C. Anisotropic dense collagen hydrogels with two ranges of porosity to mimic the skeletal muscle extracellular matrix. Biomater Adv 2023;144:213219. 36481519 10.1016/j.bioadv.2022.213219

[115] Quarta M , Cromie M, Chacon R, Blonigan J, Garcia V, Akimenko I, Hamer M, Paine P, Stok M, Shrager JB, Rando TA. Bioengineered constructs combined with exercise enhance stem cell-mediated treatment of volumetric muscle loss. Nat Commun 2017;8:15613. 28631758 10.1038/ncomms15613 PMC5481841

[116] Ansari S , Chen C, Xu X, Annabi N, Zadeh HH, Wu BM, Khademhosseini A, Shi S, Moshaverinia A. Muscle tissue engineering using gingival mesenchymal stem cells encapsulated in alginate hydrogels containing multiple growth factors. Ann Biomed Eng 2016;44:1908–20. 27009085 10.1007/s10439-016-1594-6 PMC4880526

[117] Guo B , Qu J, Zhao X, Zhang M. Degradable conductive self-healing hydrogels based on dextran-graft-tetraaniline and N-carboxyethyl chitosan as injectable carriers for myoblast cell therapy and muscle regeneration. Acta Biomater 2019;84:180–93. 30528606 10.1016/j.actbio.2018.12.008

[118] Shen C , Zhou Z, Li R, Yang S, Zhou D, Zhou F, Geng Z, Su J. Silk fibroin-based hydrogels for cartilage organoids in osteoarthritis treatment. Theranostics 2025;15:560–84. 39744693 10.7150/thno.103491 PMC11671376

[119] Li X , Li R, Wang F, Yang S, Zhou F, Hu Y, Geng Z, Su J. Theranostic hydrogels: construction strategies and applications. Chem Eng J 2025;505:159545.

[120] Li X , Sheng S, Li G, Hu Y, Zhou F, Geng Z, Su J. Research progress in hydrogels for cartilage organoids. Adv Healthc Mater 2024;13:e2400431. 38768997 10.1002/adhm.202400431

[121] Sun Y , Sheng R, Cao Z, Liu C, Li J, Zhang P, Du Y, Mo Q, Yao Q, Chen J, Zhang W. Bioactive fiber-reinforced hydrogel to tailor cell microenvironment for structural and functional regeneration of myotendinous junction. Sci Adv 2024;10:eadm7164. 38657071 10.1126/sciadv.adm7164 PMC11042749

[122] Wu X , He L, Li W, Li H, Wong W-M, Ramakrishna S, Wu W. Functional self-assembling peptide nanofiber hydrogel for peripheral nerve regeneration. Regen Biomater 2017;4:21–30. 28149526 10.1093/rb/rbw034 PMC5274702

[123] Wang Y , Wang Q, Luo S, Chen Z, Zheng X, Kankala RK, Chen A, Wang S. 3D bioprinting of conductive hydrogel for enhanced myogenic differentiation. Regen Biomater 2021;8:rbab035. 34408909 10.1093/rb/rbab035 PMC8363764

[124] Saveh-Shemshaki N , Barajaa MA, Otsuka T, Mirdamadi ES, Nair LS, Laurencin CT. Electroconductivity, a regenerative engineering approach to reverse rotator cuff muscle degeneration. Regen Biomater 2023;10:rbad099. 38020235 10.1093/rb/rbad099 PMC10676522

[125] Winston DD , Li T, Lei B. Bioactive nanoglass regulating the myogenic differentiation and skeletal muscle regeneration. Regen Biomater 2023;10:rbad059. 37492228 10.1093/rb/rbad059 PMC10365926

[126] Ronaldson-Bouchard K , Yeager K, Teles D, Chen T, Ma S, Song L, Morikawa K, Wobma HM, Vasciaveo A, Ruiz EC, Yazawa M, Vunjak-Novakovic G. Engineering of human cardiac muscle electromechanically matured to an adult-like phenotype. Nat Protoc 2019;14:2781–817. 31492957 10.1038/s41596-019-0189-8 PMC7195192

[127] Ezra E , Maor I, Bavli D, Shalom I, Levy G, Prill S, Jaeger MS, Nahmias Y. Microprocessor-based integration of microfluidic control for the implementation of automated sensor monitoring and multithreaded optimization algorithms. Biomed Microdevices 2015;17:82. 26227212 10.1007/s10544-015-9989-y

[128] Vann CG , Zhang X, Khodabukus A, Orenduff MC, Chen Y-H, Corcoran DL, Truskey GA, Bursac N, Kraus VB. Differential microRNA profiles of intramuscular and secreted extracellular vesicles in human tissue-engineered muscle. Front Physiol 2022;13:937899. 36091396 10.3389/fphys.2022.937899 PMC9452896

[129] Vesga-Castro C , Aldazabal J, Vallejo-Illarramendi A, Paredes J. Contractile force assessment methods for in vitro skeletal muscle tissues. Elife 2022;11:e77204. 35604384 10.7554/eLife.77204 PMC9126583

[130] Jeong S , Kim S, Buonocore J, Park J, Welsh CJ, Li J, Han A. A three-dimensional arrayed microfluidic blood–brain barrier model with integrated electrical sensor array. IEEE Trans Biomed Eng 2018;65:431–9. 29346110 10.1109/TBME.2017.2773463 PMC11233983

[131] Lin Q , Zhang Y, Chen L, Zhang H, An C, Li C, Wang Q, Song J, He W, Wang H. Glycine/alginate-based piezoelectric film consisting of a single, monolithic β-glycine spherulite towards flexible and biodegradable force sensor. Regen Biomater 2024;11:rbae047. 38903560 10.1093/rb/rbae047 PMC11187499

[132] Kondash ME , Ananthakumar A, Khodabukus A, Bursac N, Truskey GA. Glucose uptake and insulin response in tissue-engineered human skeletal muscle. Tissue Eng Regen Med 2020;17:801–13. 32200516 10.1007/s13770-020-00242-y PMC7710786

[133] Wang J , Zhou CJ, Khodabukus A, Tran S, Han S-O, Carlson AL, Madden L, Kishnani PS, Koeberl DD, Bursac N. Three-dimensional tissue-engineered human skeletal muscle model of Pompe disease. Commun Biol 2021;4:524. 33953320 10.1038/s42003-021-02059-4 PMC8100136

[134] Carnes ME , Pins GD. Skeletal muscle tissue engineering: biomaterials-Based strategies for the treatment of volumetric muscle loss. Bioengineering 2020;7:85. 32751847 10.3390/bioengineering7030085 PMC7552659

[135] Byun WS , Lee J, Baek J-H. Beyond the bulk: overview and novel insights into the dynamics of muscle satellite cells during muscle regeneration. Inflamm Regen 2024;44:39. 39327631 10.1186/s41232-024-00354-1 PMC11426090

[136] Carosio S , Barberi L, Rizzuto E, Nicoletti C, Prete ZD, Musarò A. Generation of eX vivo-vascularized muscle engineered tissue (X-MET). Sci Rep 2013;3:1420. 23478253 10.1038/srep01420 PMC3594753

[137] Tang Y , Wang Z, Xiang L, Zhao Z, Cui W. Functional biomaterials for tendon/ligament repair and regeneration. Regen Biomater 2022;9:rbac062. 36176715 10.1093/rb/rbac062 PMC9514853

[138] Fang Y , Han Y, Yang L, Kankala RK, Wang S, Chen A, Fu C. Conductive hydrogels: intelligent dressings for monitoring and healing chronic wounds. Regen Biomater 2025;12:rbae127. 39776855 10.1093/rb/rbae127 PMC11703555

[139] Li H , Xu X, Wu L, Chen X, Akhter H, Wang Y, Song P, Liao X, Zhang Z, Li Z, Zhou C, Cen Y, Ai H, Zhang X. Recent progress and clinical applications of advanced biomaterials in cosmetic surgery. Regen Biomater 2023;10:rbad005. 36860415 10.1093/rb/rbad005 PMC9969959

